# Unique functional responses differentially map onto genetic subtypes of dopamine neurons

**DOI:** 10.1038/s41593-023-01401-9

**Published:** 2023-08-03

**Authors:** Maite Azcorra, Zachary Gaertner, Connor Davidson, Qianzi He, Hailey Kim, Shivathmihai Nagappan, Cooper K. Hayes, Charu Ramakrishnan, Lief Fenno, Yoon Seok Kim, Karl Deisseroth, Richard Longnecker, Rajeshwar Awatramani, Daniel A. Dombeck

**Affiliations:** 1 Department of Neurobiology, Northwestern University, Evanston, IL USA; 2 Department of Neurology, Northwestern University, Chicago, IL USA; 3 Aligning Science Across Parkinson’s (ASAP) Collaborative Research Network, Chevy Chase, MD USA; 4 Department of Microbiology and Immunology, Northwestern University, Chicago, IL USA; 5 Department of Bioengineering, Stanford University School of Medicine, Stanford, CA USA; 6 Departments of Neuroscience & Psychiatry, The University of Texas at Austin, Austin, TX USA

**Keywords:** Basal ganglia, Reward, Genetics of the nervous system, Neural circuits

## Abstract

Dopamine neurons are characterized by their response to unexpected rewards, but they also fire during movement and aversive stimuli. Dopamine neuron diversity has been observed based on molecular expression profiles; however, whether different functions map onto such genetic subtypes remains unclear. In this study, we established that three genetic dopamine neuron subtypes within the substantia nigra pars compacta, characterized by the expression of *Slc17a6* (*Vglut2*), *Calb1* and *Anxa1*, each have a unique set of responses to rewards, aversive stimuli and accelerations and decelerations, and these signaling patterns are highly correlated between somas and axons within subtypes. Remarkably, reward responses were almost entirely absent in the *Anxa1*^*+*^ subtype, which instead displayed acceleration-correlated signaling. Our findings establish a connection between functional and genetic dopamine neuron subtypes and demonstrate that molecular expression patterns can serve as a common framework to dissect dopaminergic functions.

## Abstract (web summary)

The authors establish a connection between functional subtypes and genetic subtypes of dopamine neurons in mice and demonstrate that molecular expression patterns can serve as a common framework to dissect dopaminergic functions.

## Main

For decades, midbrain dopamine neurons in the substantia nigra pars compacta (SNc) and ventral tegmental area (VTA) were defined as a largely homogeneous population responding to unexpected rewards and reward-predicting cues^1–5^. However, recent studies have revealed a more complicated story, with increasing evidence for functional heterogeneity. In the VTA, dopamine neurons encode other behavioral variables, such as sensory, motor and cognitive variables, in addition to the classic reward prediction error response^6^, and separable aversive-responsive populations have been proposed^7,8^. In the SNc, dopamine neurons can respond to both rewarding and aversive stimuli^9–12^ and increase or decrease firing during movement accelerations^13–18^. Although dopamine neurons and their axons in particular regions of the SNc or striatum respond to these other behavioral variables^13,19^, reward responses have also been observed in the same regions^12,20,21^, leading to the common assumption that most, if not all, dopamine neurons robustly encode reward or reward-predicting cues. Therefore, it is currently unclear whether reward, movement and aversion encoding co-occurs in the same neurons or are separately encoded by different groups of dopamine neurons.

Diversity has also been observed in dopamine neurons at the level of gene expression. Previous limitations on the number of molecular markers that could be simultaneously studied resulted in midbrain dopamine neurons being long considered a largely homogeneous population, but recent advances in single-cell transcriptomics have led to the unbiased classification of several putative subtypes^22–27^. This leads to the enticing hypothesis that different functional responses might, in fact, map onto different molecular subtypes.

**Extended Data Fig. 1:**
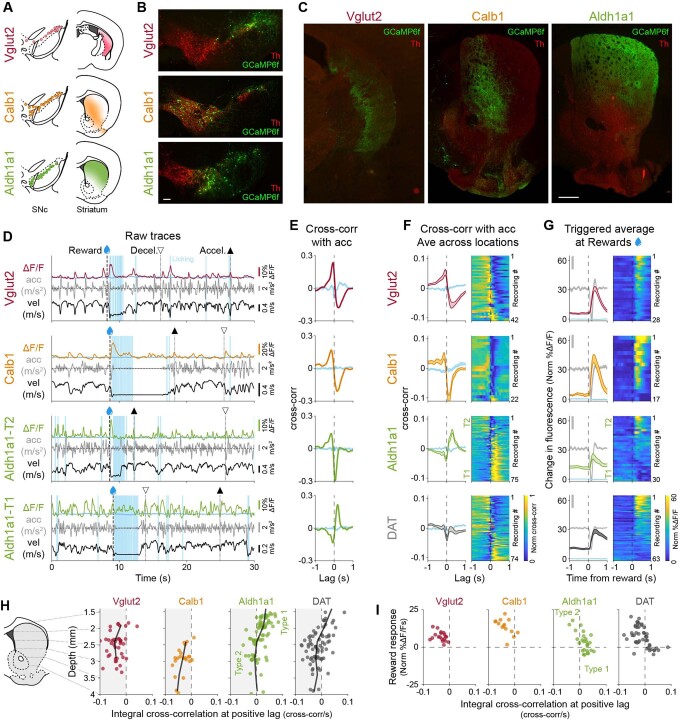
The Aldh1a1+ subtype is functionally heterogeneous. (**a**) Schematic showing the distribution of somas and axons across the SNc and striatum for three previously described subtypes (See Poulin et al. 2018^28^ for in depth characterization of each of these subtypes). (**b**) Representative distribution of somas for different subtypes within SNc. Scale bar 100 um. Thresholds for intensity scaling and gamma changes were set for each individual channel to maximize visibility of stained cells. (**c**) Representative projection patterns of different subtypes in striatum. Scale bar 500 um. Thresholds for intensity scaling and gamma changes were set for each individual channel to maximize visibility of stained axons. (**d**) Example recordings for each subtype studied (two from Aldh1a1 with different functional signaling patterns, Type 1 and Type 2), showing fluorescence traces (ΔF/F), velocity, acceleration, licking, and reward delivery times. Isosbestic control shown in blue. Large accelerations = ▲, large decelerations = ▽. (**e**) Cross-correlation between ΔF/F traces and acceleration for traces shown in D. Isosbestic control shown in blue. (**f**) Average cross-correlation between ΔF/F traces and acceleration for all recordings of each subtype and DAT (subtypes indiscriminately labeled). Isosbestic control shown in blue. Shaded regions denote mean ± s.e.m. Heatmap shows cross-correlations for each recording, sorted by the integral of the cross-correlation at positive lags. Vglut2 mice = 12, n = 42 recordings; Calb1 mice = 6, n = 22; Aldh1a1 mice = 14, n = 75 DAT mice = 14, n = 74. (**g**) ΔF/F triggered averages on reward delivery times for all recordings of each subtype and DAT. Isosbestic control shown in light blue, same scale as ΔF/F average. Acceleration shown in gray in the background (scale bar = 0.2 m/s^2^). Shaded regions denote mean ± s.e.m. Heatmap shows triggered average for each recording, sorted by size of reward response. Vglut2 mice = 11, n = 28 recordings; Calb1 mice = 8, n = 17; Aldh1a1 mice = 8, n = 30; DAT mice = 11, n = 63. (**h**) Distribution of locomotion response (integral of the cross-correlation at positive lags) along the dorso-ventral axis of the striatum for all recordings of all subtypes and DAT, showing how in Aldh1a1 dorsal recordings show acceleration correlation (Type 1) while more ventral recordings show deceleration correlation (Type 2). Black line represents moving average (0.5 mm bins). (**i**) Relationship between reward response and locomotion response for each recording of each subtype, showing how in Aldh1a1 larger reward responses correspond with deceleration correlation (Type 2), while small or negative reward responses correspond with acceleration correlation (Type 1). Source data

In this Article, we address this question with a focus on the SNc. Three different subtypes have been proposed to account for most of the SNc dopamine neurons^22^, which we here refer to by marker genes that characterizes each subtype: the *Aldh1a1*^+^, *Calb1*^+^ and *Vglut2*^+^ (which is also enriched in *Calb1* expression) subtypes. These subtypes have somas in SNc which, although intermingled, are anatomically biased: *Aldh1a1*^*+*^ somas are biased toward ventral SNc, *Calb1*^*+*^ somas toward dorsal SNc and *Vglut2*^*+*^ somas toward lateral SNc^28^ (Extended Data Fig. 1a,b). Similarly, their axons project to different regions of striatum although with overlap in some regions: *Aldh1a1*^*+*^ axons project to dorsal and lateral striatum, *Calb1*^*+*^ to dorso-medial and ventro-medial striatum and *Vglut2*^*+*^ most densely to posterior striatum^28^ (Extended Data Fig. 1a,c). If these different subtypes indeed have different functional signaling properties, their anatomical biases might explain previous seemingly conflicting results showing different functional responses of dopamine neurons during the same behaviors^13–21,29^: different subtype(s) may have been inadvertently investigated based on the recording location in SNc or striatum.

## Results

To functionally characterize the different dopamine neuron subtypes, we used intersectional genetic strategies (Methods) to isolate three known SNc genetic subtypes (*Aldh1a1*^+^, *Vglut2*^+^ and *Calb1*^+^) and label them with the calcium indicator GCaMP6f^30^. We then used fiber photometry to record GCaMP calcium transients from groups of striatal axons of the isolated dopaminergic subtypes in head-fixed mice running on a treadmill while periodically receiving unexpected rewards or aversive stimuli (air puffs to the face, which have been shown to cause avoidance in mice^10^). These simple experimental manipulations were designed to test the involvement of different subtypes in the most commonly studied roles of dopamine, movement, aversion and reward and to allow comparisons with as wide a range of existing research as possible. To control for any movement artifacts, we also recorded GCaMP fluorescence at its isosbestic wavelength, 405 nm^30^. GCaMP is ideally suited for these experiments because all known mechanisms for triggering axonal dopamine release involve increases in intracellular calcium concentration^31,32^, including anterogradely propagating action potentials and cholinergic modulation^33,34^. Critically, the detected calcium transients are generated only from the labeled genetic subtypes; non-labeled neurons do not contribute. For this reason, GCaMP is preferred here over extracellular dopamine sensors (that is, dLight, GRAB-DA and microdialysis) because axons from different subtypes can overlap in many striatal regions^28^, and these sensors detect dopamine released from all nearby axons, without subtype specificity.

We will expand and describe the functional signaling properties of the different genetic subtypes in detail in subsequent sections. First, however, we describe a discovery that we made about the *Aldh1a1*^*+*^ subtype that prompted us to refine the current genetic classification of dopamine neurons. Given the selective loss of *Aldh1a1* dopamine neuron staining in Parkinson’s disease^35,36^, we expected that this subtype might show acceleration-correlated responses^13^. Functional recordings, however, revealed clear functional heterogeneity across the different recording locations from *Aldh1a1*^*+*^ axons (Extended Data Fig. 1d–i). *Aldh1a1*^*+*^ axons projecting to dorsal striatum displayed acceleration-correlated signaling and no detectable response to rewards (termed a ‘Type 1’ functional response), whereas axons projecting more ventrally displayed deceleration-correlated signaling and responded robustly to rewards (‘Type 2’) (Extended Data Fig. 1h,i). This functional heterogeneity was markedly different from recordings from the *Calb1*^*+*^ and *Vglut2*^*+*^ subtypes, which were largely homogenous across recording locations with deceleration-correlated signaling and a robust reward response (similar to *Aldh1a1*^+^ ‘Type 2’). The *Aldh1a1* Type 1 response was remarkable, in that it suggested that there might exist a dopamine neuron subtype that did not respond to rewards and, instead, showed acceleration-correlated responses. If true, this would contradict the notion that all dopamine neurons robustly signal reward. Thus, the functional heterogeneity that we observed within *Aldh1a1*^*+*^ recordings motivated us to reexamine the existing dopamine neuron classification schemes and search for new genetic subtypes within the SNc *Aldh1a1*^*+*^ population with such signaling patterns.

### *Anxa1*^+^, a new subtype within *Aldh1a1*^+^

**Extended Data Fig. 2:**
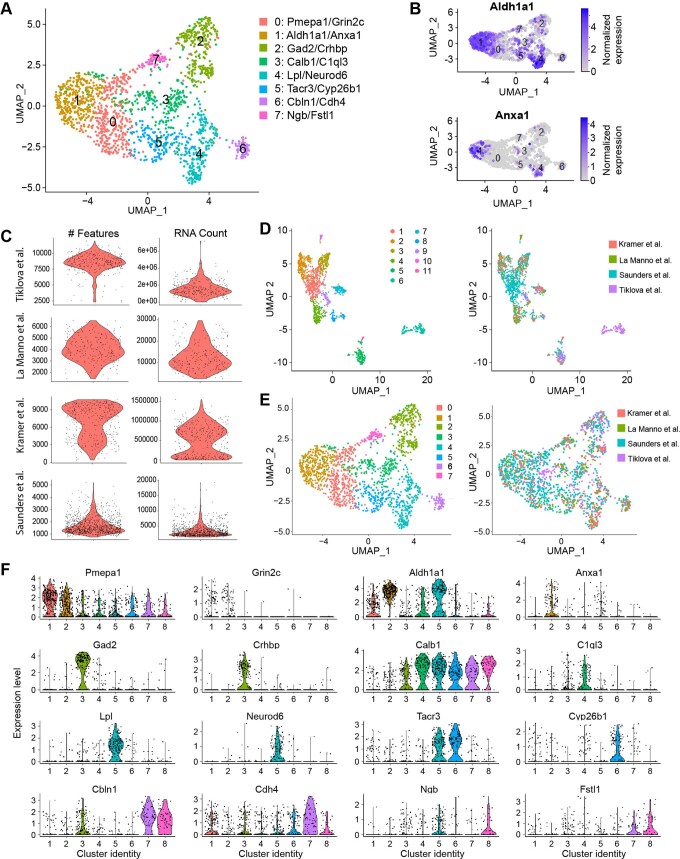
Integration of scRNA-seq datasets reveals more granular resolution of DA neuron subtypes. (**a**) Resulting clusters from integrating datasets. (**b**) Expression patterns of *Anxa1* and *Aldh1a1*, the top defining markers for cluster 1. Expression of *Anxa1* appears to be limited to a subset of *Aldh1a1*-expressing neurons. (**c**) Violin plots of number of genes and RNA counts from each source dataset, which were used to determine cutoffs for quality control filtering. (**d**) LIGER clustering of the meta-dataset, revealing one cluster that was more distantly related to all other DA neurons and came solely from the Tiklova et al.^26^ dataset. This cluster was subsequently removed. (**e**) Cells colored by cluster (left) or source dataset (right), which reveals that all clusters were represented by each dataset. (**f**) Violin plots of the top 2 defining marker genes for each cluster.

The current classification of dopamine neurons was derived through single-cell gene expression profiling, primarily via single-cell RNA sequencing (scRNA-seq)^22^. However, such studies are limited by the number of cells analyzed due to technical difficulties in scRNA-seq, which could lead to inconclusive identification of closely related clusters. To uncover more granular divisions among dopaminergic subtypes, we first combined the data from four scRNA-seq studies^24–27^ into an unbiased meta-dataset (Methods). We observed eight clusters, one of which was defined by co-expression of *Aldh1a1* and *Anxa1* (Extended Data Fig. 2a). These markers were previously shown to co-localize to ventral SNc subtypes^24,37^, and plotting the expression of these two genes showed that *Anxa1* expression is limited to a subset of *Aldh1a1*^*+*^ neurons (Extended Data Fig. 2b). This raised the possibility of at least two molecularly distinct *Aldh1a1*^*+*^ populations. However, although the analysis of this meta-dataset was able to refine our mapping of dopaminergic neuron subtypes, it was still limited by the biases introduced by the individual source datasets and cross-dataset integration methods and, thereby, necessitated further validation.

**Fig. 1:**
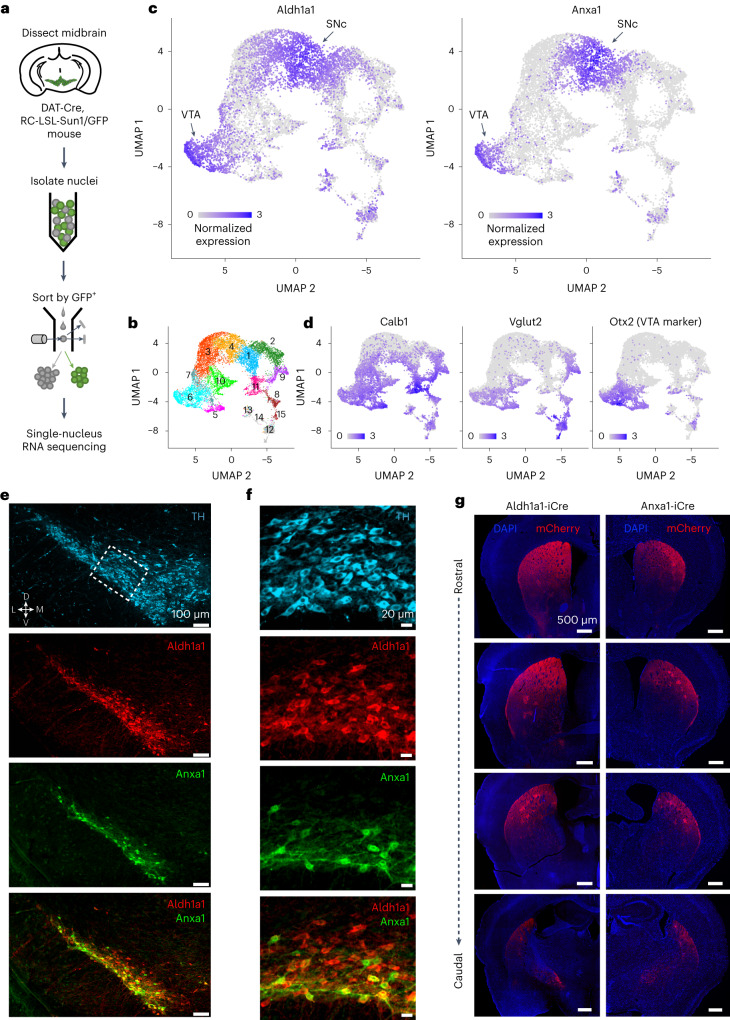
snRNA-seq reveals an *Anxa1*-expressing subtype within *Aldh1a1*^+^ dopamine neurons. **a**, Schematic of snRNA-seq experimental pipeline. **b**, UMAP reduction of resulting clusters. In total, 15 clusters were found. Notably, four clusters (12, 13, 14 and 15) had weak dopaminergic characteristics (see Extended Data Fig. 3 for details). **c**, Expression of *Aldh1a1* and *Anxa1*, the latter of which is expressed only within a subset of *Aldh1a1*-expressing neurons. **d**, Expression patterns of the additional markers used for genetic access in experiments here, as well as *Otx2*, a classical marker of most VTA neurons, enriched in clusters 5, 6 and 7. **e**, Immunofluorescence images of *Aldh1a1* and *Anxa1* protein expression in SNc (*n* = 4 mice). Anxa1 expression is limited to a ventral subset of *Aldh1a1*^+^ neurons. Thresholds for intensity scaling and gamma changes were set for each individual channel to maximize visibility of stained cells. **f**, Zoomed-in crops of section shown in **e**. Anxa1 expression was ventrally biased within SNc neurons. **g**, Right, projection patterns of *Anxa1*^+^ SNc axons based on viral labeling (*n* = 4 mice), which appear highly restricted to dorsolateral striatum and patches. Left, projection patterns of *Aldh1a1*^+^ SNc axons using the same virus (*n* = 4 mice); projections extend more ventrally relative to *Anxa1*^+^. Maximum thresholds for image intensity scaling were set to the highest detected pixel intensity in each section to better enable direct comparisons across brains. Source data

**Extended Data Fig. 3:**
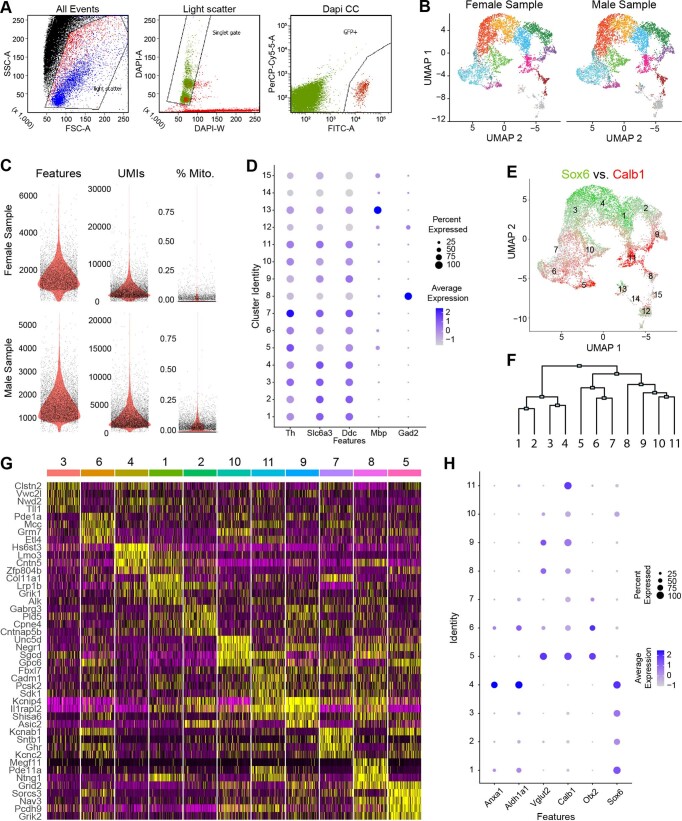
Details of generation and analysis of single-nucleus RNAseq dataset. (**a**) Example plots from FACS sorting of GFP+ nuclei. (**b**) Plots showing the distribution of cells from either the male or female samples, showing all clusters were represented by both samples. (**c**) Quality control plots of number of genes (features), UMIs, and percent mitochondrial reads for each sample. (**d**) Dotplot of classic DA neuron markers as well as *Mbp* and *Gad2*, which were used to determine non-classical DA clusters (12, 13, 14 & 15). Clusters 12, 14 & 15 significantly under-express DA neuron markers. *Mbp* is significantly expressed in cluster 13. *Gad2* expression is limited to cluster 8, suggesting this cluster represents a previously described population of gabaergic dopamine neurons. (**e**) Overlaid expression patterns of *Sox6* (green) and *Calb1* (red) recapitulates a previously observed dichotomy among midbrain dopamine neurons. (**f**) Dendrogram of hierarchical clustering estimation. Height of branch points approximates the relatedness of clusters. Notably, clusters 1–4 appear to be *Sox6*+, 5–7 are *Otx2*+, and 8–11 are negative for both markers. (**g**) Heatmap of top 4 differentially expressed genes for each cluster, excluding non-classical DA clusters. (**h**) Dotplot of expression of key marker genes of dopamine neuron subpopulations.

**Extended Data Fig. 4:**
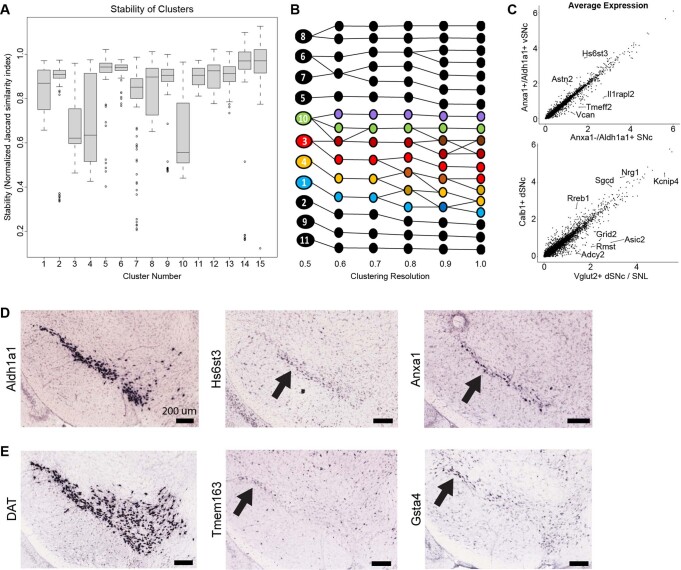
Cluster heterogeneity and distinguishing features. (**a**) Quantification of stability (via normalized Jaccard similarity index) of all 15 clusters from n = 100 iterations of stability calculations (simulated randomly down-sampled datasets, see Methods). Lower stability measurements imply the possibility of further subdivisions within the cluster, or additional subpopulations that may have been split across adjacent clusters. Center represents median, upper and lower box bounds represent 75^th^ and 25^th^ percentiles respectively, whiskers represent maxima and minima excluding outliers (data points more than 1.5 times the IQR outside the box bounds). (**b**) Mapping clusters across progressively higher resolutions reveals potential subdivisions either within clusters (for example, the splitting of Cluster 8 into two stable clusters) or across adjacent clusters (for example a novel cluster emerging at the intersection of clusters 3 and 10 as resolution increases). Four clusters with lower stability, as shown in panel A, are colored to highlight the potential source of their instabilities. (**c**) Scatter plots comparing the average expression for all genes across two clusters. Several examples of distinguishing genes with notably enriched expression patterns are highlighted. Top: Clusters 4 (*Anxa1*+/*Aldh1a1*+) vs. 1 (*Anxa1*-/*Aldh1a1*+). Bottom: Clusters 11 (*Calb1*+ SNc) vs. 9 (*Vglut2*+ SNc/SNL). Transcriptomic similarity of cluster pairs can be approximated by the correlation coefficient of their average gene expressions. (**d**) In situ hybridization images from the Allen Mouse Brain Atlas of ventral tier marker genes. Note that *Hs6st3*, which is highly enriched in our *Anxa1*+ cluster, appears limited to ventral-most SNc and highly resembles the expression of Anxa1 (black arrows). Images available from Allen Mouse Brain Atlas, mouse.brain-map.org (**e**) Additional ISH images showing expression of two marker genes that distinguish Cluster 9 (*Vglut2*+) from Cluster 11 (*Calb1*+), further corroborating the distinct identities of these populations. *DAT* expression is shown for reference to highlight the localization of these markers to SN pars lateralis, matching the previously described location of *Vglut2*+ SN DA neurons and thus supporting Cluster 9 as the *Vglut2*+ neurons investigated in the GCaMP activity recordings in this study. Source images available from Allen Mouse Brain Atlas, mouse.brain-map.org.

To overcome the technical limitations of single-cell isolation of dopamine neurons, we used single-nucleus gene profiling (snRNA-seq), a technique that is more efficient in brain regions where the recovery of intact neurons is difficult^38^. Indeed, this strategy allowed us to profile over 12,000 dopaminergic neuron nuclei from five DAT-Cre, CAG-Sun1/sfGFP mice (Fig. 1a), an order of magnitude higher than previous single-cell studies^23–27^. This approach resulted in the unbiased identification of 15 clusters, out of which four minor clusters (12–15, colored in gray in Fig. 1b and Extended Data Fig. 3) represent neurons with weak dopaminergic characteristics (Methods). The remaining clusters show expression profiles largely in agreement with previous reports from single-cell sequencing studies but with further subdivision of clusters. Notably, all clusters were represented in both male and female samples (Extended Data Fig. 3b). Three clusters (1, 3 and 4) were significantly enriched for Sox6 (Wilcoxon rank-sum test, false discovery rate (FDR)-adjusted *P* values = 4.6 × 10^−150^, 9.8 × 10^−66^ and 2.8 × 10^−276^, respectively). Cluster 2 also showed enrichment of Sox6 (*P* = 1.1 × 10^−4^); however, this result did not survive FDR correction. Four clusters (5, 6, 9 and 11) were significantly enriched for *Calb1* (FDR-adjusted *P* values = 6.6 × 10^−30^, 1.7 × 10^−4^, 1.1 × 10^−22^ and 1.5 × 10^−71^, respectively). Cluster 10 also showed *Calb1* enrichment (*P* = 6.9 × 10^−4^), which, again, did not survive FDR correction. Little overlap between these genes was observed (Extended Data Fig. 3e), recapitulating a fundamental dichotomy among dopaminergic neurons^23,39^. Furthermore, *Vglut2* expression is mainly limited to a subset of *Calb1*^*+*^ cells (Fig. 1d and Extended Data Fig. 3h), consistent with prior recombinase-based labeling experiments^28^. Based on the expression patterns of these genes, as well as other differentially expressed markers (Extended Data Fig. 4c–e and Methods), we infer that clusters 9 and 11 represent the SNc *Vglut2*^*+*^ and *Calb1*^*+*^ subtypes from which calcium transients were recorded, respectively. We also observed two likely SNc clusters with high *Aldh1a1* expression (1 and 4; Fig. 1c and Extended Data Fig. 3h). The third *Aldh1a1*^*+*^ cluster (6) was *Sox6*^*−*^ and *Otx2*^+^ (Fig. 1c and Extended Data Fig. 3e) and corresponds to a previously described VTA subtype also expressing *Aldh1a1*^+^ (ref. ^28^). Cluster 4 was, again, significantly enriched for *Anxa1* expression (FDR-adjusted *P* = 9.4 × 10^−118^) (Fig. 1c), corroborating the results from our integrated dataset analysis and establishing *Anxa1* as a discrete dopamine neuron subtype marker within *Aldh1a1*^*+*^ neurons.

**Extended Data Fig. 5:**
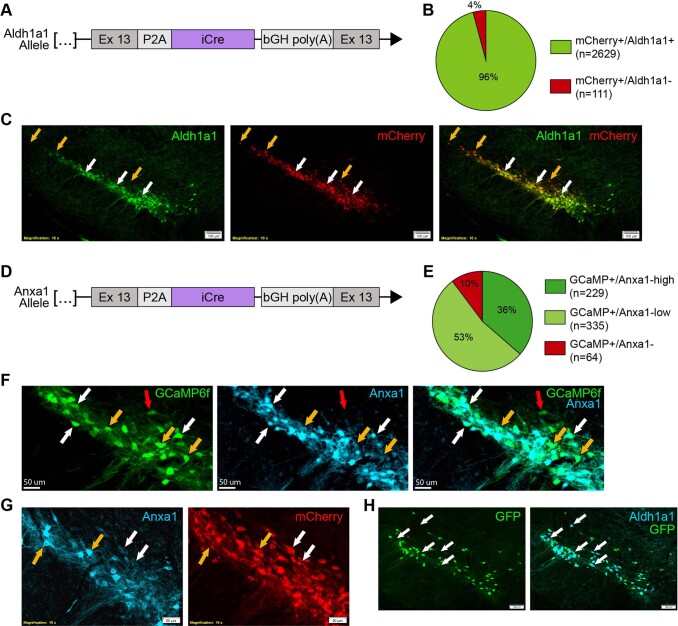
(**a**) Schematic representation of Aldh1a1-iCre transgenic line. Endogenous Aldh1a1 gene was targeted for insertion of a P2A peptide and iCre immediately following the peptide encoded by Exon 13. (**b**) Ratio of mCherry virally labelled cells co-staining for Aldh1a1 (n = 4 mice). (**c**) Substantia nigra pars compacta immunofluorescence staining from Aldh1a1-iCre mice injected with an AAV5-DIO-mCherry virus (n = 4 mice). Co-staining shows excellent efficiency and fidelity of iCre recombination, which is notably limited to TH+ cells in this region. White arrows: examples of mCherry and Aldh1a1 co-stained cells. Orange arrows: mCherry-expressing cells with undetectable Aldh1a1 staining, which were primarily localized to the dorsal and lateral SNc. Thresholds for intensity scaling and gamma changes were set for each individual channel to maximize visibility of stained cells. (**d**) Schematic representation of Anxa1-iCre transgenic line. (**e**) Ratios of virally labelled cells co-staining for Anxa1 protein (n = 4 mice), showing high fidelity of Cre recombination. (**f**) High magnification of immunofluorescence staining from Anxa1-iCre mice injected with an AAV1-CAG-FLEX-GCaMP6f virus (n = 4 mice) shows that recombination occurs in cells with both high Anxa1 (white arrow) and low Anxa1 (orange arrow), with ~10% of labelled cells showing undetectable Anxa1 protein (red arrows). Thresholds for intensity scaling and gamma changes were set for each individual channel to maximize visibility of stained cells. (**g**) High magnification of immunofluorescence staining from Anxa1-iCre mice injected with an AAV5-DIO-mCherry virus (n = 4 mice) confirms that recombination occurs in cells with both high Anxa1 protein staining (orange arrows) as well as low Anxa1 protein (white arrows), making it difficult to assess specificity using protein staining alone. Thresholds for intensity scaling and gamma changes were set for each individual channel to maximize visibility of stained cells. (**h**) IF staining of GFP and Aldh1a1 in Anxa1-iCre, TH-Flpo, RC::FrePe mice (n = 2 mice). Recombination by iCre and Flpo leads to GFP expression in Anxa1+ DA neurons. Co-staining with Aldh1a1 corroborates that Anxa1-iCre recombination is less broad than Aldh1a1 expression and confirms that viral labelling results were not due to insufficient viral delivery / diffusion (example cells with Aldh1a1 staining but no recombination shown with white arrows). Thresholds for intensity scaling and gamma changes were set for each individual channel to maximize visibility of stained cells. Source data

After the identification of *Anxa1*^*+*^ as a putative subtype marker, immunostaining confirmed that SNc neurons expressing *Anxa1* protein are indeed part of the broader *Aldh1a1*^+^ population and, in fact, have cell bodies located ventrally within the already ventral *Aldh1a1*^+^ region (Fig. 1e,f). We, thus, generated a new mouse line, Anxa1-iCre, to genetically access this subtype (Methods and Extended Data Fig. 5d–h). This allowed us to observe the axonal arbors of *Anxa1*^*+*^ dopamine neurons, which, in comparison to *Aldh1a1* axon arbors, innervate a more dorsally restricted region of the striatum (Fig. 1g). Notably, this projection pattern matched the observed anatomical distribution of *Aldh1a1*^+^ ‘Type 1’ axons, suggesting that these unique functional responses could map onto the *Anxa1*^*+*^ subtype.

### Genetic subtypes show different signaling patterns during locomotion

**Fig. 2:**
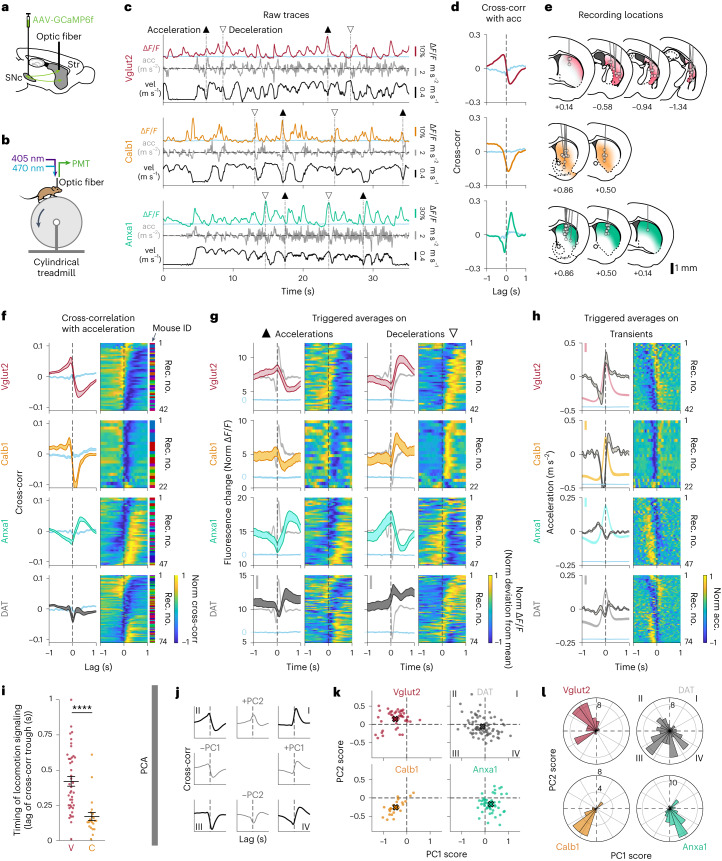
Dopaminergic genetic subtypes display different signaling patterns during locomotion. **a**, Strategy used to label dopamine neuron subtypes and record from their axons in striatum with GCaMP6f, a calcium indicator whose changes in fluorescence can be used as a proxy for neuronal firing. **b**, Schematic of fiber photometry recording setup. **c**, Example recordings from each subtype studied, showing fluorescent traces (Δ*F*/*F*), mouse acceleration and velocity. Isosbestic control shown in blue. ▲, large accelerations; ▽, large decelerations. **d**, Cross-correlation between Δ*F*/*F* traces and acceleration for traces shown in **c**. Isosbestic control shown in blue. **e**, Recording locations in striatum for recordings shown in **f**–**h**. Shaded colors represent projection patterns for each subtype. **f**, Average cross-correlation between Δ*F*/*F* traces and acceleration for all recordings of each subtype and DAT (subtypes indiscriminately labeled). Isosbestic control shown in blue. Shaded regions denote mean ± s.e.m. across recordings. Heat map shows cross-correlation for each recording, sorted by PC1/PC2 angle (see **l**). Vglut2 mice = 12, *n* = 42 recordings; Calb1 mice = 6, *n* = 22 recordings; Anxa1 mice = 10, *n* = 47 recordings; DAT mice = 14, *n* = 74 recordings. See Extended Data Fig. 6i for averages per mouse. **g**, Δ*F*/*F* averages triggered on large accelerations (left, ▲) and large decelerations (right, ▽) for all recordings of each subtype and DAT. Isosbestic control shown in blue, same scale as Δ*F*/*F* average but shifted for visibility. Acceleration shown in gray in background (scale bar, 0.2 m s^−^^2^). Shaded regions denote mean ± s.e.m. across recordings. Heat map shows triggered average for each recording, sorted as in **f**. **h**, Acceleration averages triggered on Δ*F*/*F* transient peaks for all recordings of each subtype and DAT. Δ*F*/*F* average and isosbestic control shown in background (bar, 5% normalized Δ*F*/*F*). Shaded regions denote mean ± s.e.m. across recordings. Heat map shows triggered average for each recording, sorted as in **f**. **i**, Timing analysis showing the lag of the trough in the Δ*F*/*F*-acceleration cross-correlations for each recording from Calb1 and Vglut2, as shown in **f** (same recordings and *n*). Mean Vglut2 = 0.42, Calb1 = 0.17; *P* value for comparison = 1 × 10^−6^ (two-sided Wilcoxon rank-sum test with Bonferroni correction, same *n* as **f**). Error bars denote mean ± s.e.m. Analogous analysis conducted for triggered averages in Extended Data Fig. 6f,g. **j**–**l**, PCA conducted on Δ*F*/*F*-acceleration cross-correlations for all striatal recordings from Vglut2, Calb1 and Anxa1 subtypes. **j**, ±PC1 and ±PC2 loadings (gray) and their combinations (black), which represent the different quadrants shown in **k**–**l**. Together, PC1 and PC2 account for 84.3% of variance of all cross-correlations (PC1 = 64.2% of variance, PC2 = 20.1%). **k**, PC scores for each recording of each subtype and DAT along PC1 and PC2. X shows mean for each subtype. **l**, Radial histogram showing the PC1/PC2 angle of each recording in **k**. *P* values for comparison between subtypes VC = 2 × 10^−7^, VA = 4 × 10^−11^ and CA = 2 × 10^−4^ (two-sided Wilcoxon rank-sum test with Bonferroni correction). Acc, acceleration; Cross-corr, cross-correlation; Rec. no., recording number. Source data

To functionally characterize the different dopamine neuron subtypes during locomotion, we used genetic strategies (Methods) to isolate *Vglut2*^+^, *Calb1*^+^ and *Anxa1*^*+*^ subtypes (as well as *Aldh1a1*^*+*^ for comparison and *DAT* mice where all subtypes were indiscriminately labeled). We then used fiber photometry to record GCaMP calcium transients from populations of striatal axons of isolated dopaminergic subtypes (~300-µm-diameter volumes sampled across the striatal projection regions) in head-fixed mice running on a treadmill (Fig. 2a,b). Because the *Vglut2*^*+*^ subtype is contained within *Calb1*^+^, in our *Calb1*^*+*^ recordings we avoided recording from the posterior striatum where *Vglut2*^*+*^ neurons project; thus, our *Calb1*^*+*^ recordings come largely from *Calb1*^+^*/Vglut2*^*−*^ neurons, which project to the medial striatum (Extended Data Fig. 1a–c).

**Fig. 3:**
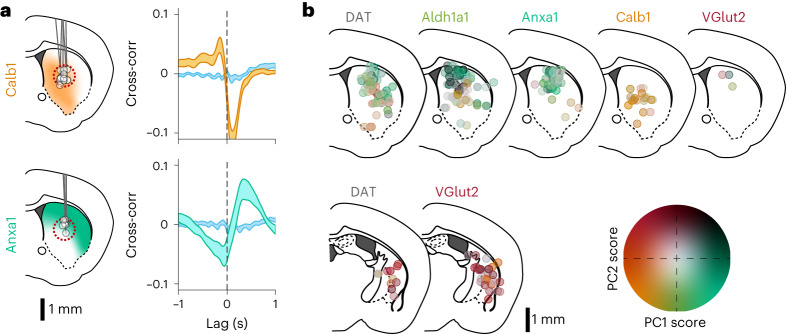
Spatial distribution of subtype-specific locomotion responses. **a**, Comparison of locomotion response (cross-correlation between Δ*F*/*F* and acceleration) for Calb1 and Anxa1 recordings only from a region of striatum where their axons overlap, dashed red circle (1-mm diameter). Isosbestic controls in blue. Shaded areas denote mean ± s.e.m. Calb1 mice = 4, *n* = 5 recordings; Anxa1 mice = 5, *n* = 16 recordings. **b**, Locomotion response (PC1/PC2 angle, as shown in Fig. 2l) mapped onto recording location for each subtype and DAT. Locations from the body (top) or the tail of the striatum (bottom) were collapsed into a single brain section. To reduce overlap, locations were shifted a random amount between ±0.4 mm mediolaterally. See Extended Data Fig. 6a for an expanded version of this panel without shifts or collapsing slices together. Cross-corr, cross-correlation. Source data

**Extended Data Fig. 6:**
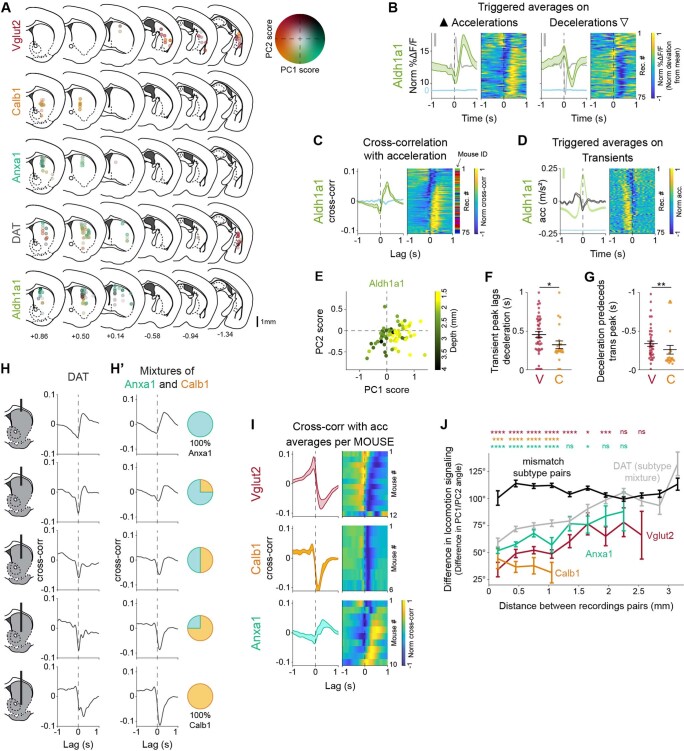
Dopaminergic genetic subtypes show different signaling patterns during locomotion. (**a**) Locomotion response (PC1/PC2) mapped onto recording location for each subtype and DAT. Same as Fig. 3b but without collapsing slices for compactness and without random mediolateral shifting of recording locations to reduce overlap. (**b**) Average ΔF/F triggered on large accelerations (left, ▲) and large decelerations (right, ▽) for Aldh1a1 recordings (as Fig. 2g). Isosbestic control shown in light blue, same scale as ΔF/F average but shifted. Acceleration shown in grey in the background (scale bar = 0.2 m/s^2^). Shaded regions denote mean ± s.e.m. Heatmap shows triggered average for each recording, sorted by PC1/PC2 angle (see Fig. 2l). Aldh1a1 mice = 14, n = 75 recordings. (**c**) Average cross-correlation between ΔF/F traces and acceleration for Aldh1a1 recordings (as Fig. 2f). Isosbestic control shown in blue. Shaded regions denote mean ± s.e.m. Heatmap shows cross-correlation for each recording, sorted as in B. (**d**) Average acceleration triggered on large transients for Aldh1a1 recordings (as Fig. 2h). ΔF/F average and isosbestic control shown in the background (scale bar = 5% Norm ΔF/F.) Shaded regions denote mean ± s.e.m. Heatmap shows triggered average for each recording, sorted as in B. (**e**) Principal component scores for each recording along PC1 and PC2 for Aldh1a1 (same as Fig. 2k) but with each Aldh1a1 recording color-coded by depth within striatum, showing that Aldh1a1 axons deeper in striatum show similar locomotion signaling to Calb1. (**f**) Timing of the calcium transient peak in triggered averages on decelerations (Fig. 2g, right) for each recording from Calb1 and Vglut2. Means Vglut2 = 0.35, Calb1 = 0.23; p-value for comparison between subtypes = 0.01 (two-sided Wilcoxon rank-sum test). Vglut2 mice = 12, n = 42 recordings; Calb1 mice = 6, n = 22 (as in Fig. 2i). Error bars denote mean ± s.e.m. (**g**) Timing of the deceleration peak in triggered averages on ΔF/F transient peaks (Fig. 2h) for each recording from Calb1 and Vglut2. Means Vglut2 = 0.47, Calb1 = 0.34; p-value for comparison between subtypes = 0.005 (two-sided Wilcoxon rank-sum test). Same n as F. Error bars denote mean ± s.e.m. (**h**) The locomotion signaling observed in DAT mice across depths (H) can be explained by mixtures of the Anxa1 and Calb1 subtypes in varying ratios matching the relative abundance of each subtypes’ axons in that depth (H’). (**i**) Average cross-correlation between ΔF/F traces and acceleration for all recordings of each functionally homogeneous subtype (as Fig. 2f) but averaged per mouse. Shaded regions denote mean ± s.e.m. Heatmap shows cross-correlation average for each mouse, sorted by PC1/PC2 angle (see Fig. 2l). (**j**) Difference in locomotion signaling (measured as the difference in PC1/2 angle, as shown in Fig. 2l) between pairs of recordings made at difference distances from each other, for pairs of recordings from the same subtype (Vglut2, Calb1, and Anxa1, in colors), from DAT mice (mixture of subtypes, in grey), or from mismatched subtypes (Vglut2-Calb1, Vglut2-Anxa1, and Calb1-Anxa1). P-values for comparison between pairs within same subtype vs mismatch subtypes: Vglut2 = 3 × 10^−05^, 6 × 10^−23^, 2 × 10^−25^, 5 × 10^−25^, 1 × 10^−08^, 0.03, 9 × 10^−04^, 1, 0.9; Calb1 = 2 × 10^−04^, 5 × 10^−22^, 4 × 10^−10^, 1 × 10^−07^; Anxa1 = 8 × 10^−08^, 6 × 10^−35^, 1 × 10^−21^, 5 × 10^−09^, 0.06, 0.02, 1, 1 (two-sided Mann-Whitney U test with Bonferroni correction). Number of pairs of recordings per distance bin (from 0 in steps of 0.3 mm): Vglut2 = [24, 107, 108, 91, 58, 29, 28, 14, 6, 0, 0], Calb1 = [37, 83, 29, 18, 1, 3, 0, 0, 0, 0, 0], Anxa1 = [252, 410, 250, 52, 49, 30, 28, 10, 0, 0, 0], Mismatch = [47, 245, 438, 525, 461, 661, 542, 615, 367, 189, 49]. Error bars denote mean ± s.e.m. Source data

**Extended Data Fig. 7:**
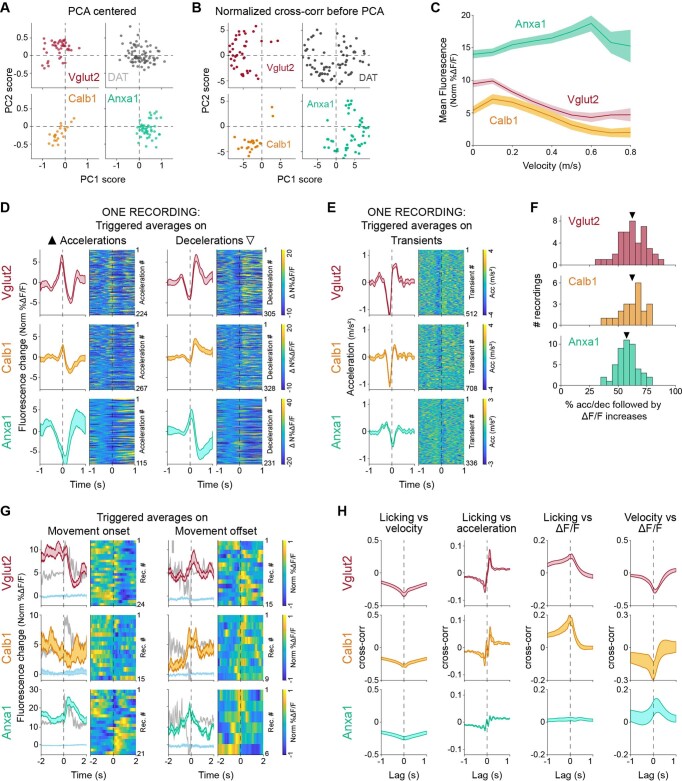
Dopaminergic genetic subtypes show different signaling patterns during locomotion continued. (**a**) Principal component scores for each recording of each subtype and DAT along PC1 and PC2, as show in Fig. 2k but when PCA analysis is conducted with data centering, showing this variable does not have an effect in the differentiation of subtypes. (**b**) Principal component scores for each recording of each subtype and DAT along PC1 and PC2, as show in Fig. 2k but where the cross-correlations for each recording have been min-max scaled before running PCA analysis. This has a similar effect as considering only the angle of PC1/2 as shown in Fig. 2l, with recordings being pushed out into an annulus of which different subtypes occupy different sectors. (**c**) Ongoing velocity is encoded by each subtype. Average fluorescence for each subtype at different ranges of velocities (bin width 0.1 m/s). Error bands denote mean ± s.e.m. (**D**) Average ΔF/F triggered on large accelerations (left, ▲) and large decelerations (right, _) (as Fig. 2g) for one representative recording of each subtype. Shaded regions denote mean ± s.e.m. across events. Heatmap shows ΔF/F traces for each acceleration or deceleration for the recording in chronological order, shifted to better show relative changes in fluorescence (subtract average ΔF/F in a window −0.7 to −0.1 s before trigger points). (**e**) Average acceleration triggered on large transients (as Fig. 2h) for one representative recording of each subtype. Shaded regions denote mean ± s.e.m. across events. Heatmap shows acceleration traces for each transient in the recording in chronological order. (**f**) Percent of accelerations (for Anxa1) or decelerations (for Vglu2 and Calb1) followed by an increase in ΔF/F. Mean: Vglut2 = 62.4%, Calb1 = 62.3%, Anxa1 = 57.5%. (**g**) Average ΔF/F triggered on movement onsets (left) and offsets (right) for each subtype. Isosbestic control shown in light blue, same scale as ΔF/F average but shifted. Acceleration shown in grey in the background (scale bar = 0.5 m/s^2^). Shaded regions denote mean ± s.e.m. across recordings. Heatmap shows ΔF/F traces for each recording randomly sorted. Mice: Vglut2 = 10/8, Calb1 = 5/8, Anxa1 = 8/4. (**h**) Cross-correlations between different behavioral variables. Shaded regions denote mean ± s.e.m. Source data

Remarkably, we observed distinct functional responses in dopamine neuron subtypes. *Calb1*^*+*^ and *Vglut2*^*+*^ axons preferentially signaled during locomotion decelerations, whereas *Anxa1*^*+*^ axons preferentially signaled during locomotion accelerations (Fig. 2c), similarly to *Aldh1a1*^+^ ‘Type 1’ (Extended Data Figs. 1d–f and 6b–e). Accordingly, cross-correlations between calcium Δ*F*/*F* traces (Δ*F*/*F* traces) and acceleration revealed a deep trough at positive time lags for *Calb1*^*+*^ and *Vglut2*^*+*^ axons (indicative of calcium transient peaks after decelerations), but a large peak at positive lags for *Anxa1*^*+*^ axons (transient peaks after accelerations; Fig. 2d), and this was consistent across a wide range of striatum locations (Fig. 2e,f). The opposing signaling patterns of *Calb1*^*+*^ and *Vglut2*^*+*^ versus *Anxa1*^*+*^ were also clear in Δ*F*/*F* averages triggered on accelerations or decelerations (Fig. 2g), acceleration averages triggered on Δ*F*/*F* transient peaks (Fig. 2h) and Δ*F*/*F* averages triggered on movement onsets and offsets (Extended Data Fig. 7g), as well as in their relationship with velocity (Extended Data Fig. 7c). Furthermore, these signaling differences persist even in regions where axons from different subtypes overlap (Fig. 3a, b) and as a function of recording distance (pairs of recordings from the same subtype displayed higher similarity in locomotion signaling than pairs from different subtypes; Extended Data Fig. 6j), together indicating that these functional differences were intrinsic to each subtype and were not simply defined by striatal projection location. In contrast, in *DAT* mice where subtypes were indiscriminately labeled, heterogeneous signaling was observed across striatal recording locations (Fig. 2f–h, bottom, and, to a lesser extent, in *Aldh1a1*^+^; Extended Data Fig. 6b–d).

Interestingly, signaling differences were also evident between *Vglut2*^*+*^ and *Calb1*^*+*^ in their timing with respect to decelerations, with *Calb1*^*+*^ transients after decelerations with a shorter lag than *Vglut2*^*+*^ (Fig. 2i and Extended Data Fig. 6f,g). To further quantify such differences, we used a dimensionality reduction technique to extract the components that best explain the variance in the cross-correlations. We applied principal component analysis (PCA) to the matrix of all cross-correlation traces from *Vglut2*^+^, *Calb1*^+^ and *Anxa1*^*+*^ subtypes (Methods), finding that the first two principal components (PC1 and PC2) explained 84.3% of the variance in the cross-correlations (64.2% PC1 and 20.1% PC2). We observed that different combinations of PC1 and PC2 closely approximated the cross-correlation averages of the different subtypes: PC1^+^ + PC2^−^ for *Anxa1*^+^, PC1^−^ + PC2^−^ for *Calb1*^+^and PC1^−^ + PC2^+^ for *Vglut2*^+^ (Fig. 2j,h). Accordingly, the decomposition of each recording along these PCs revealed distributions that were well separated between the subtypes (Fig. 2k,l; mean PC1/PC2 angles, representing the timecourse of the cross-correlations and, thus, the temporal relationship between Δ*F*/*F* and acceleration = 141° for *Vglut2*^+^, 208° for *Calb1*^+^ and 239° for *Anxa1*^+^, *P* values = 2 × 10^−7^ V–C, 4 × 10^−11^ V–A and 2 × 10^−4^ C–A, Wilcoxon rank-sum test with Bonferroni correction). Cross-correlations from *DAT* recordings decomposed using the same PCs were spread across the same regions of the PC1/PC2 space as individual subtypes and areas in between (Fig. 2k, l, dark gray, and, to a lesser extent, in *Aldh1a1*^+^; Extended Data Fig. 6e). These different decompositions of *DAT* (and *Aldh1a1*^+^) recordings also mapped onto different striatal locations (Fig. 3b and Extended Data Fig. 6a). *DAT* recordings that displayed similar decomposition to a particular subtype (for example, dorsal striatum to *Anxa1*^*+*^ or posterior striatum to *Vglut2*^+^) suggest that a single subtype dominated *DAT* signaling within the photometry recording volume in these striatal regions. However, the *DAT* recordings that displayed a different mixture of PCs than any particular subtype (for example, middle depth striatum) suggest that a mixture of subtype axons were contained within the recording volume (Fig. 3b and Extended Data Fig. 6a). In fact, *DAT*’s signaling pattern across depths within striatum could be explained by modeling combinations of *Calb1*^*+*^ and *Anxa1*^*+*^ in different ratios, approximating the relative abundance of axons from these subtypes at each depth (Extended Data Fig. 6h).

Overall, these findings demonstrate that, during locomotion, *Calb1*^+^, *Vglut2*^*+*^ and *Anxa1*^*+*^ dopamine neuron subtype axons displayed different average functional signaling patterns. *Calb1*^*+*^ and *Vglut2*^*+*^ axons were largely deceleration correlated with unique timing differences between these subtypes, whereas *Anxa1*^*+*^ axons were largely acceleration correlated.

### Subtypes show different responses to rewards and aversive stimuli

**Fig. 4:**
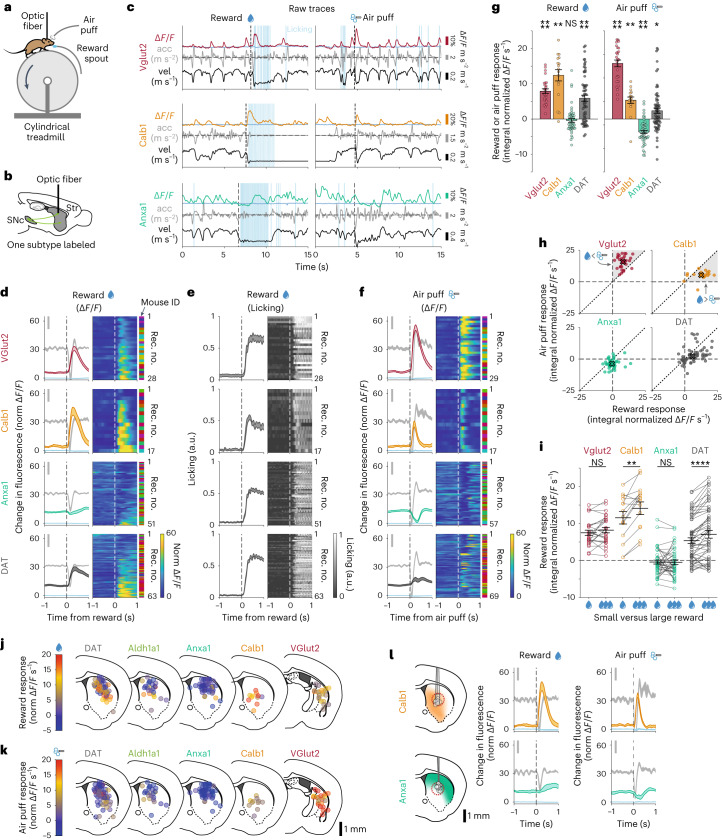
Dopaminergic genetic subtypes display different responses to rewards and aversive stimuli. **a**, Mouse running on treadmill during fiber photometry while receiving unexpected rewards and air puffs. **b**, Schematic of fiber photometry recording strategy. **c**, Example recordings for each subtype studied, showing fluorescence traces (Δ*F*/*F*), mouse velocity, acceleration, licking and reward (left) or air puff (right) delivery times. Isosbestic controls in light blue, same scale as Δ*F*/*F* traces. Reward and air puff examples for each subtype are from the same recording. **d**, Δ*F*/*F* averages triggered on reward delivery times for all recordings of each subtype and DAT. Isosbestic control in light blue, same scale as Δ*F*/*F* average. Acceleration shown in gray in background (scale bar, 0.2 m s^−^^2^). Shaded regions denote mean ± s.e.m. across recordings. Heat maps show triggered average for each recording, sorted by size of reward response. Vglut2 mice = 11, *n* = 28 recordings; Calb1 mice = 8, *n* = 17 recordings; Anxa1 mice = 8, *n* = 51; DAT mice = 11, *n* = 63 recordings. See Extended Data Fig. 8h,i for averages per mouse. **e**, Licking average triggered on reward delivery times for all recordings of each subtype and DAT (same as **d**). Shaded areas denote mean ± s.e.m. across recordings. Heat map shows triggered average for each recording, sorted as in **d**. **f**, Δ*F*/*F* averages triggered on air puff delivery times for all recordings of each subtype and DAT. Isosbestic control in light blue, same scale as Δ*F*/*F* average. Acceleration shown in gray in background (scale bar, 0.2 m s^−^^2^). Shaded regions denote mean ± s.e.m. Heat map shows triggered average for each recording, sorted by reward size as in **d**,**e**. Vglut2 mice = 12, *n* = 29 recordings; Calb1 mice = 8, *n* = 17 recordings; Anxa1 mice = 8, *n* = 57 recordings; DAT mice = 11, *n* = 69 recordings. **g**, Average reward and air puff responses for each subtype (integral of fluorescence in a 0.5-s window after stimulus minus integral in 0.5 s before stimulus). Error bars denote mean ± s.e.m. across recordings. Means (m) and *P* values for reward: Vglut2 mice = 7.9 normalized Δ*F*/*F* s, *P* = 2 × 10^−5^; Calb1 mice = 12.4, *P* = 0.001; Anxa1 mice = −0.5, *P* = 0.1 (NS); DAT mice = 5.9, *P* = 9 × 10^−7^. Means (m) and *P* values for air puff: Vglut2 mice = 15.8, *P* = 1 × 10^−5^; Calb1 mice = 5.3, *P* = 0.007, Anxa1 mice = −3.7, *P* = 4 × 10^−8^; DAT mice = 5.3, *P* = 0.02 (two-sided Wilcoxon signed-rank test with Bonferroni correction). Same *n* as **d**,**f**. **h**, Reward versus air puff responses for all recordings of each subtype and DAT. X shows mean for each subtype. Shaded regions are areas representing greater air puff than reward response (for Vglut2) or greater reward versus air puff response (for Calb1). **i**, Comparison of responses to small versus large rewards for each subtype. Error bars denote mean ± s.e.m. Mean difference (m) and *P* values: Vglut2 mice = 0.9 normalized Δ*F*/*F* s, *P* = 0.6 (NS); Calb1 mice = 3.9, *P* = 9 × 10^−3^; Anxa1 mice = 0.04, *P* = 1 (NS); DAT mice = 1.9, *P* = 8 × 10^−5^(two-sided paired Wilcoxon signed-rank test with Bonferroni correction). Vglut2 mice = 11, *n* = 25 recordings; Calb1 mice = 6, *n* = 14 recordings; Anxa1 mice = 8, *n* = 42 recordings; DAT mice = 10, *n* = 55 recordings. **j**, Reward response mapped onto recording locations for each subtype and DAT. Locations from the body or the tail of the striatum were collapsed into a single brain section. To reduce overlap, locations were shifted a random amount between ±0.4 mm mediolaterally. See Extended Data Fig. 8j,k for an expanded version of this panel without shifts or collapsing slices together. **k**, Same as **j** but for air puff response. **l**, Comparison of reward and air puff response for Calb1 and Anxa1 recordings only from a region of striatum where their axons overlap, dashed red circle. Isosbestic control in blue. Shaded regions denote mean ± s.e.m. across recordings. Calb1 mice = 4, *n* = 9 recordings; Anxa1 mice = 5, *n* = 13 for rewards, *n* = 17 for air puffs. acc, acceleration; vel, velocity. Source data

**Fig. 5:**
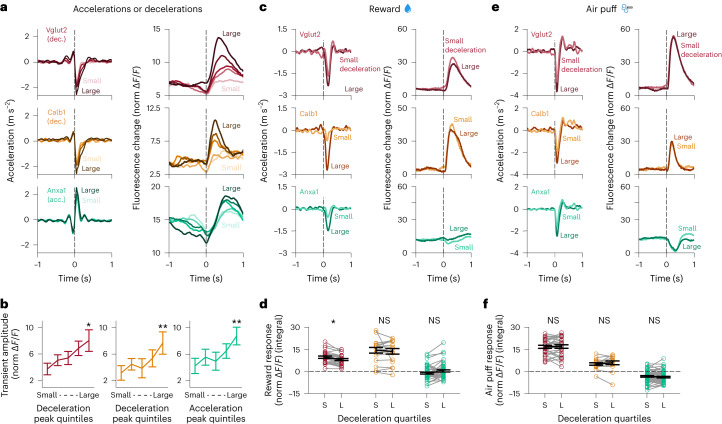
Transients scale with acceleration/deceleration amplitude, but reward and air puff responses are independent of movement. **a**, Acceleration (left) and Δ*F*/*F* (right) averages triggered on decelerations (for Vglut2 and Calb1) and accelerations (for Anxa1, bottom) as in Fig. 2g but with decelerations/accelerations split into five quantiles based on their amplitude. Vglut2 mice = 12, *n* = 42 recordings; Calb1 mice = 6, *n* = 22 recordings; Anxa1 mice = 10, *n* = 47 recordings (same as Fig. 2f–h). **b**, Average Δ*F*/*F* transient amplitude for decelerations/accelerations of increasing size, as shown in **a**. Percent increase in transient amplitude for the largest versus smallest quintile of decelerations/accelerations: Vglut2 = 214%, Calb1 = 243%, Anxa1 = 206%. *P* values: Vglut2 = 0.01, Calb1 = 0.002, Anxa1 = 0.002 (two-sided paired Wilcoxon signed-rank test with Bonferroni correction). Same *n* as **a**. Error bars denote mean ± s.e.m. **c**, Acceleration (left) and Δ*F*/*F* (right) averages triggered on rewards as in Fig. 4d but split into two quantiles based on the amplitude of the accompanying deceleration. Vglut2 mice = 11, *n* = 28 recordings; Calb1 mice = 8, *n* = 17 recordings; Anxa1 mice = 8, *n* = 51 recordings (same as Fig. 4d).¸**d**, Average Δ*F*/*F* transient amplitude for rewards based on the size of the accompanying deceleration, as shown in **c**. *P* values for comparison between transient amplitude for the smallest versus largest decelerations: Vglut2 = 0.01 (but for a decrease in transient amplitude for larger decelerations), Calb1 = 1 (NS) and Anxa1 = 0.1 (NS) (two-sided paired Wilcoxon signed-rank test with Bonferroni correction). Same *n* as **c**. Error bars denote mean ± s.e.m. **e**, Acceleration (left) and Δ*F*/*F* (right) averages triggered on air puffs as in Fig. 4f but split into two quantiles based on the amplitude of the accompanying deceleration. Vglut2 mice = 12, *n* = 29 recordings; Calb1 mice = 8, *n* = 17 recordings; Anxa1 mice = 8, *n* = 57 recordings (same as Fig. 4f). **f**, Average Δ*F*/*F* transient amplitude for air puffs based on the size of the accompanying deceleration, as shown in **e**. *P* values for comparison between transient amplitude for the smallest versus largest decelerations: Vglut2 = 1 (NS), Calb1 = 1 (NS) and Anxa1 = 1 (NS) (two-sided paired Wilcoxon signed-rank test with Bonferroni correction). Same *n* as **e**. Error bars denote mean ± s.e.m. Source data

**Extended Data Fig. 8:**
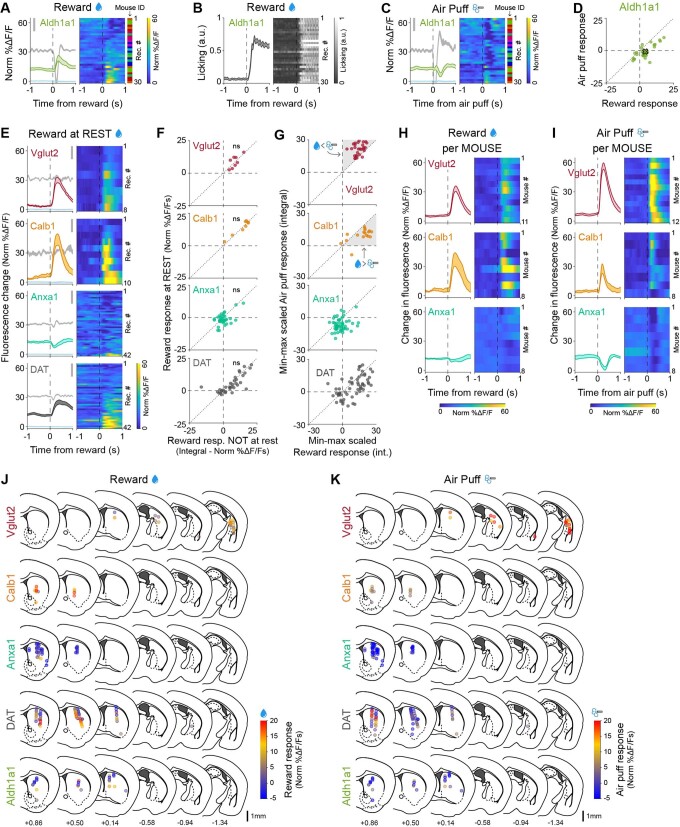
Dopaminergic subtypes show different signaling to rewards and aversive stimuli. (**a**) ΔF/F average triggered on reward delivery times for all recordings from Aldh1a1 (as Fig. 4d). Isosbestic control shown in light blue, same scale as ΔF/F average. Acceleration shown in gray in the background (scale bar = 0.2 m/s^2^). Shaded regions denote mean ± s.e.m. Heatmap shows triggered average for each recording, sorted by size of reward response. Aldh1a1 mice = 8, n = 30 recordings. (**b**) Licking average triggered on reward delivery times (same as A) for all recording from Aldh1a1 (as Fig. 4e). Shaded regions denote mean ± s.e.m. Heatmap shows triggered average for each recording, sorted as in A. (**c**) ΔF/F average triggered on air puff delivery times for all recordings from Aldh1a1 (as Fig. 4f). Isosbestic control shown in light blue, same scale as ΔF/F average. Acceleration shown in gray in the background (scale bar = 0.2 m/s^2^). Shaded regions denote mean ± s.e.m. Heatmap shows triggered average for each recording, sorted by reward size as in A, B. Aldh1a1 mice = 8, n = 30 recordings. (**d**) Reward vs air puff responses for Aldh1a1 (as shown in Fig. 4h for other subtypes). X shows mean. (**e**) ΔF/F averages triggered on rewards delivered during rest for all recordings of each subtype and DAT. Isosbestic control shown in light blue, same scale as ΔF/F average. Acceleration shown in gray in the background (scale bar = 0.2 m/s^2^). Shaded regions denote mean ± s.e.m. Heatmaps show triggered average for each recording, sorted by size of the reward response. Vglut2 mice = 6, n = 8 recordings; Calb1 mice = 6, n = 10; Anxa1 mice = 8, n = 42; DAT mice = 10, n = 42. (**f**) Comparison between response to rewards at rest (E) vs response to rewards not at rest for all recordings of each subtype and DAT. Diagonal dotted line represents identity line (same response to rewards at rest vs all rewards). p-values: Vglut2 = 1 (ns), Calb1 = 1 (ns), Anxa1 = 0.4 (ns), DAT = 0.2 (ns), two-sided paired Wilcoxon signed-rank test with Bonferroni correction. (**g**) Subtypes can still be distinguished by their air puff and reward responses after min-max scaling the responses. (**H**) ΔF/F average triggered on reward delivery times for all recordings of each functionally homogeneous subtype (as Fig. 4d) but averaged per mouse. Shaded regions denote mean ± s.e.m. Heatmap shows triggered average for each recording, randomly sorted. (**i**) Same as H but for air puffs. (**j**) Reward response mapped onto recording locations for each subtype and DAT. Same as Fig. 4j but without collapsing slices for compactness and without random mediolateral shifting of recording locations to reduce overlap. (**k**) Same as J but for air puff response (matching Fig. 4k but without collapsing slices or random shifting). Source data

We then asked whether these dopaminergic subtypes respond differently to rewards and aversive stimuli. We randomly delivered unexpected water rewards and aversive air puffs to the whiskers/face to mice already habituated to run on the treadmill (Fig. 4a) and used fiber photometry to record Δ*F*/*F* transients from populations of axons at different striatal locations (Fig. 4b). We found that both *Calb1*^*+*^ and *Vglut2*^*+*^ axons responded robustly to rewards (Fig. 4c–e,g; *P* = 2 × 10^−5^ and *P* = 0.001, respectively, Wilcoxon signed-rank test with Bonferroni correction) and air puffs (Fig. 4c,f,g; *P* = 1 × 10^−5^ and *P* = 0.007, respectively) consistently across nearly all recording locations. The reward signaling in *Calb1*^*+*^ and *Vglut2*^*+*^ axons could not be explained by their movement responses during reward delivery, as the amplitude of the reward signals was the same at rest (Extended Data Fig. 8e,f; *P* = 1 (not significant (NS)) for both *Vglut2*^*+*^ and *Calb1*^+^, paired Wilcoxon signed-rank test with Bonferroni correction). Furthermore, there was no increase in the size of the reward responses to larger decelerations (Fig. 5c,d), whereas the amplitude of non-reward-associated decelerations did correlate with the size of the transient in *Vglut2*^*+*^ and *Calb1*^*+*^ (Fig. 5a,b; *Vglut2*^*+*^ 214% change, *P* = 0.01 and *Calb1*^*+*^ 243% change, *P* = 0.002; paired Wilcoxon signed-rank test with Bonferroni correction). As for air puffs, there was, again, no significant increase in air puff responses to larger decelerations (Fig. 5e,f). Interestingly, although the *Vglut2*^*+*^ and *Calb1*^*+*^ subtype axons both responded to rewards and air puffs, their responses still differed. *Vglut2*^*+*^ axons displayed larger responses to air puffs than reward, whereas *Calb1*^*+*^ axons displayed larger responses to rewards than air puffs (Fig. 4h). Furthermore, *Calb1*^*+*^ axons displayed larger responses to increased reward size—a hallmark of reward prediction error (RPE) and value coding^40^ (Fig. 4i). This response increase was not detectable from *Vglut2*^*+*^ axons.

In contrast to *Calb1* and *Vglut2* axons, however, unexpected reward responses were almost entirely absent from *Anxa1*^*+*^ axons (Fig. 4c–e,g; integral of response: mean = −0.5, *P* = 0.1, NS), but they did respond to air puffs with a signaling decrease (Fig. 4c,f,g; integral of response: mean = −3.7, *P* = 4 × 10^−8^). Again, this air puff response was not explained by mouse movement, as the amplitude of the decrease was not modulated by deceleration (Fig. 5e,f), whereas, in contrast, the amplitude of non-air-puff-associated accelerations did correlate with the size of the transient in *Anxa1*^*+*^ axons (Fig. 5a,b; 206% change, *P* = 0.002, paired Wilcoxon signed-rank test with Bonferroni correction). Notably, these differences persist even in regions where axons from different subtypes may overlap (Fig. 4l), indicating that they were intrinsic to each subtype and not simply defined by striatal projection location. The reward and air puff responses from *DAT* recordings were location specific, with few reward responses in dorsal striatum but responses more prevalent in more ventral and posterior regions (Fig. 4j,k).

Thus, these results further highlight the functional diversity within these subtypes: *Vglut2*^*+*^ axons displayed a greater response to aversive stimuli than rewards, and *Calb1*^*+*^ axons displayed a greater response to rewards than aversive stimuli and were robustly sensitive to reward size, whereas reward responses were largely undetectable from *Anxa1*^*+*^ axons, which, instead, displayed a signaling decrease to aversive stimuli.

### Dopamine neuron functions differentially map onto genetic subtypes

**Fig. 6:**
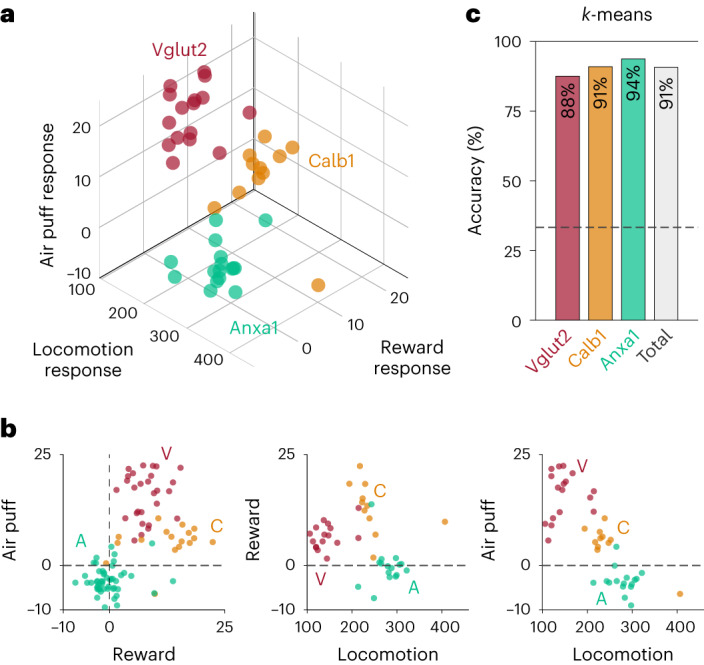
Unique locomotion, reward and air puff responses differentially map onto genetic subtypes of dopamine neurons. **a**, 3D plot showing locomotion (PC1/PC2 angle; Fig. 2l), reward and air puff responses for each recording and each subtype. **b**, 2D plots corresponding to all the combinations of the three dimensions used in **a**. **c**, Unsupervised *k*-means classification distinguished subtypes based on locomotion (PC1 and PC2 scores), reward and air puff responses, with total accuracy of 91%: 14/16 Vglut2, 10/11 Calb1 and 15/16 Anxa1 recordings correctly classified. Dashed line represents chance accuracy (33%). Source data

To explicitly demonstrate the connection between functional and genetic dopamine neuron subtypes, we plotted the locomotion signaling (PC1/PC2 angles from Fig. 2l), response to rewards and response to air puffs for the subset of recordings where all three measurements were made (Fig. 6a,b). *Calb1*^+^, *Vglut2*^*+*^ and *Anxa1*^*+*^ recordings resided in separable regions of this three-dimensional (3D) functional space, with minimal overlap. This separation is maintained even when the triggered averages and cross-correlations are scaled to ignore their amplitude and consider only their timecourse (Extended Data Figs. 7b and 8g). We then asked whether an unsupervised classification method, *k*-means clustering, could distinguish the subtypes based on these functional dimensions. Indeed, when searching for three clusters within the functional space, *k*-means separated the recordings into clusters that matched the genetic subtypes with 91% accuracy (Fig. 6c; of note, random chance = 33% accuracy, 88% accuracy for *Vglut2*^+^, 91% for *Calb1*^+^ and 94% for *Anxa1*^+^). Thus, our findings establish a clear connection between functional responses and genetic dopamine neuron subtypes and demonstrate that genetically defined subtypes of nigrostriatal dopamine axons have, on average within a small recording volume, markedly different signaling patterns during locomotion, reward and aversive stimuli.

### Axons track somatic signaling within subtypes

Slice studies have shown that coordinated activation of striatal cholinergic interneurons can not only modulate but also trigger dopamine release in the absence of somatic firing^33,34,41,42^. A pioneering in vivo study recently provided strong support for the idea that this local mechanism plays a substantial role in dopamine release during behavior^43^. This study found that dopamine release from striatal axons co-varied with reward expectation, whereas firing in the midbrain somas did not, and further observed fast striatal dopamine release during certain behavioral epochs that did not correspond with somatic firing. However, establishing that dopamine is released from axons independently of somatic firing in vivo requires that axonal and somatic recordings are made from the same neurons^44^. Thus, an alternative explanation for any observed soma–axon signaling differences is that the striatal dopamine detected was released by a different set of axons than those belonging to the recorded somas—an experimental recording problem that could be rectified by labeling and recording from only one genetic subtype at a time.

**Extended Data Fig. 9:**
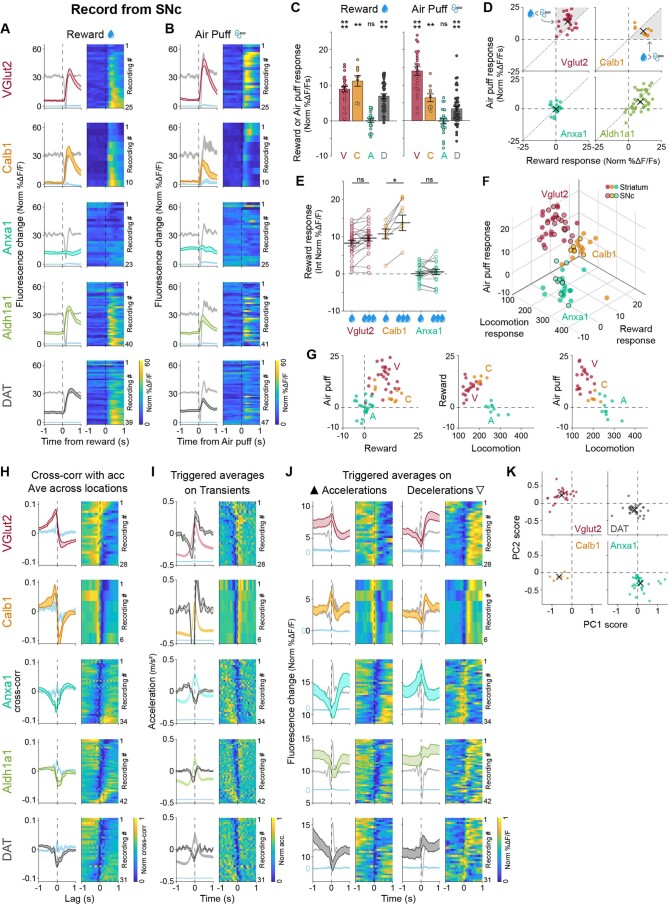
SNc somas of genetic dopamine neuron subtypes have similar signaling patterns to their axons in response to rewards and air puffs and during locomotion. (**a**-**e**) Same as Fig. 4 but for recordings made in SNc. **(a)** ΔF/F averages triggered on reward delivery times for all recordings of each subtype and DAT. Isosbestic control shown in light blue, same scale as ΔF/F average. Acceleration shown in gray in the background (scale bar = 0.2 m/s 2). Shaded regions denote mean ± s.e.m. Heatmap shows triggered average for each recording, sorted by size of reward response. Vglut2 mice = 9, n = 25 recordings; Calb1 mice = 5, n = 10; Anxa1 mice = 5, n = 23; Aldh1a1 mice = 11, n = 40; DAT mice = 7, n = 39. **(b)** ΔF/F averages triggered on air puff delivery times for all recordings of each subtype and DAT. Isosbestic control shown in light blue, same scale as ΔF/F average. Acceleration shown in gray in the background (scale bar = 0.2 m/s 2). Shaded regions denote mean ± s.e.m. Heatmap shows triggered average for each recording, sorted by reward size as in A. Vglut2 mice = 9, n = 25 recordings; Calb1 mice = 5, n = 10; Anxa1 mice = 5, n = 25; Aldh1a1 mice = 11, n = 41; DAT mice = 8, n = 47. **(c)** Average reward and air puff responses for each subtype. Error bars denote ± s.e.m. p-values for reward: Vglut2 = 5 × 10^−05^, Calb1 = 0.007, Anxa1 = 1 (not significant), DAT = 2 × 10^−07^. p-values for air puff: VGlut2 = 5 × 10^−05^, Calb1 = 0.008, Anxa1 = 1 (not significant), DAT = 3 × 10^−05^. Two-sided Wilcoxon sign-rank test with Bonferroni correction. Same recordings and n as A,B. **(d)** Reward vs air puff responses for all recordings of each subtype and DAT. X shows mean for each subtype. Shaded regions are areas representing greater air puff than reward response (for Vglut2) or greater reward vs air puff response (for Calb1). **(e)** Comparison of responses to small vs large rewards for each subtype. Error bars denote mean ± s.e.m. p-values: Vglut2 = 0.05 (not significant), Calb1 = 0.03, Anxa1 = 1 (not significant). Two-sided paired Wilcoxon Signed Rank test with Bonferroni correction. Vglut2 mice = 9, n = 25 recordings; Calb1 mice = 5, n = 10; Anxa1 mice = 5, n = 23. **(F)** 3D plot showing locomotion (PC1/PC2 angle), reward and air puff responses for each recording and each subtype, comparing striatal recordings (same as Fig. 6a) and SNc recordings. **(G)** 2D plots for each pair of variables shown in the 3D plot in F. (**h**-**k**) Same as Fig. 2 but for recordings made in SNc. (**h**) Average cross-correlation between ΔF/F traces and acceleration for all recordings of each subtype. Isosbestic control shown in blue. Shaded regions denote mean ± s.e.m. Heatmap shows cross-correlation for each recording, sorted by PC1/PC2 angle (see Fig. 2l). Vglut2 mice = 11, n = 28 recordings; Calb1 mice = 3, n = 6; Anxa1 mice = 8, n = 34; Aldh1a1 mice = 13, n = 42; DAT mice = 8, n = 31. (**I**) ΔF/F averages triggered on large accelerations (left, ▲) and large decelerations (right, ▽) for all recordings of each subtype. Isosbestic control shown in light blue, same scale as ΔF/F average but shifted. Acceleration shown in gray in the background (scale bar = 0.2 m/s^2^). Shaded regions denote mean ± s.e.m. Heatmap shows triggered average for each recording, sorted as in H. (**J**) Acceleration averages triggered on large transients for all recordings of each subtype. ΔF/F average and isosbestic control shown in the background (scale bar = 5% Norm ΔF/F.) Shaded regions denote mean ± s.e.m. Heatmap shows triggered average for each recording, sorted as in H. (**K**) Principal component scores for each recording of each subtype along PC1 and PC2 (same PCs obtained from the striatal recordings, as shown in Fig. 2j–l). X shows mean for each subtype. Striatal PCs explain 77.6% of SNc variance (52.4% PC1, 25.2% PC2). Source data

Given the functional differences that we observed in axons of different subtypes, we first asked whether the somas of these same subtypes show similar functional signaling as their axons. We repeated the above photometry recording experiments but placed the optic fiber in SNc instead of striatum. At somas, GCaMP transients are caused by somatic action potential firing. Indeed, just as in the axonal recordings, *Calb1*^*+*^ and *Vglut2*^*+*^ somas responded to rewards and air puffs, whereas reward responses were largely undetectable in *Anxa1*^*+*^ somas (Extended Data Fig. 9a–f). *Calb1*^*+*^ somas also showed greater responses to rewards than air puffs, and *Vglut2*^*+*^ somas, on average, had greater responses to air puffs than rewards (Extended Data Fig. 9c,d), similarly to their axons. *Calb1*^*+*^ somas also showed greater responses to larger rewards (Extended Data Fig. 9e). Furthermore, soma recordings from each of the subtypes showed highly similar signaling during locomotion compared to axons (Extended Data Fig. 9g–k) and fell into the same, separable regions of the 3D functional space as axonal recordings of the same subtypes (Extended Data Fig. 9f). Thus, axons and somas of the same dopamine neuron subtype displayed highly similar signaling correlations to locomotion and responses to rewards and aversive stimuli. This is further evidence that functional responses map onto genetic subtypes, as somas of individual subtypes intermingled to a fair degree in SNc, particularly within the photometry recording volume.

**Fig. 7:**
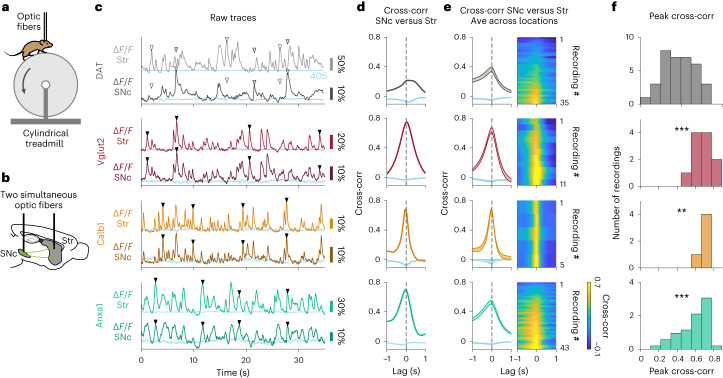
Highly correlated signaling in axons and somas within genetic subtypes of dopamine neurons. **a**, Mouse running on treadmill during dual fiber photometry. **b**, Schematic of simultaneous photometry recordings from SNc and striatum. **c**, Example recordings for DAT and each subtype showing simultaneous fluorescence traces (Δ*F*/*F*) from SNc and striatum. Isosbestic controls in blue. ▼, example transients present in SNc and in striatum; ▽, example transient present in striatum but not in SNc (white fill) or vice versa (gray fill). **d**, Cross-correlation between Δ*F*/*F* traces from striatum and SNc shown in **c**. Isosbestic controls in blue. **e**, Average cross-correlation between simultaneous Δ*F*/*F* traces from striatum and SNc for all recordings of each subtype and DAT. Isosbestic controls in blue. Shaded regions denote mean ± s.e.m. across recordings. Heat map shows cross-correlations for each paired recording sorted by peak magnitude. DAT mice = 5, *n* = 35 recordings; Vglut2 mice = 4, *n* = 11 recordings; Calb1 mice = 2, *n* = 5 recordings; Anxa1 mice = 8, *n* = 43 recordings. **f**, Distribution of peak cross-correlations between SNc and striatum for recordings of all subtypes and DAT shown in **e**. *P* values for comparison to DAT: Vglut2 = 3 × 10^−4^, Calb1 = 3 × 10^−3^, Anxa1 = 3 × 10^−4^ (two-sided Mann–Whitney *U*-test with Bonferroni correction). Ave, average; Cross-corr, cross-correlation; Str, striatum. Source data

**Extended Data Fig. 10:**
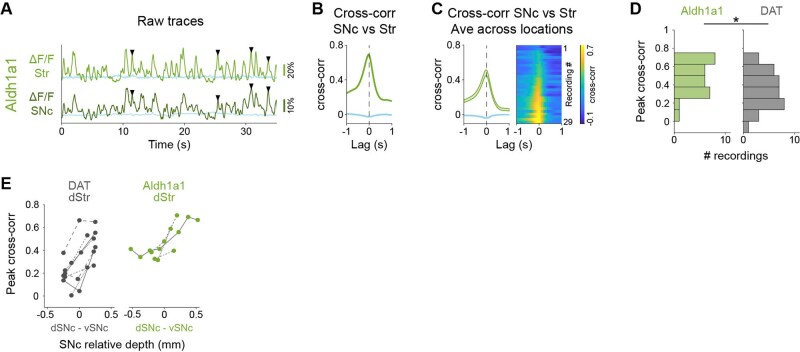
Highly correlated signaling in axons and somas within genetic subtypes of dopamine neurons. (**a**) Example recording for Aldh1a1 showing simultaneous fluorescence traces (ΔF/F) from SNc and striatum. Isosbestic control shown in blue. ▼= Example transients present in SNc and in striatum. (**b**) Cross-correlation between ΔF/F traces from striatum and SNc shown in A. Isosbestic control shown in blue. (**c**) Average cross-correlation between ΔF/F traces from striatum and SNc for all recordings of Aldh1a1 (as Fig. 6e). Isosbestic control shown in blue. Shaded regions denote mean ± s.e.m. Heatmap shows cross correlations for each paired recording sorted by peak magnitude. Aldh1a1 mice = 8, n = 29 recordings. (**d**) Distribution of peak cross correlations between SNc and striatum for recordings of Aldh1a1 and DAT shown in C (as Fig. 6f). P-value for comparison DAT-Aldh1a1 = 0.03 (two-sided Mann-Whitney U test with Bonferroni correction). (**e**) Peak cross correlations between dorsal striatum recordings (from Aldh1a1 or DAT) vs different relative depths in SNc, showing that for Aldh1a1 dorsal striatum signaling is best correlated to ventral SNc. Source data

However, it is still possible that somas and axons could have similar correlation to movements or stimuli but low correlations to each other (for example, somas and axons could be active at different accelerations or stimuli). Therefore, we performed simultaneous striatal axon and SNc soma recordings. Before recording from dopamine neuron subtypes, we first asked whether we could reproduce the soma–axon signaling differences previously described in non-subtype-specific recordings^43^ but with GCaMP and in head-fixed mice running on a treadmill. We labeled non-subtype-specific dopamine neurons (DAT-Cre mice) and used fiber photometry to simultaneously record from populations of axons in the striatum with one fiber and SNc somas with another fiber (Fig. 7a,b). We recorded from a range of random locations within striatum and SNc and often observed highly dissimilar signaling (Δ*F*/*F*) between striatal axons and SNc somas (Fig. 7c and Extended Data Fig. 10a for *Aldh1a1*^+^). Accordingly, the mean cross-correlation between axonal and somatic Δ*F*/*F* traces (Fig. 7d,e) was 0.37, which is a relatively low correlation for traces that have similar temporal dynamics (in contrast to cross-correlations between Δ*F*/*F* and acceleration, which have dissimilar temporal dynamics; Fig. 2f). Therefore, similarly to previous reports^43^, we found somatic and axonal dopamine neuron signaling that was often very different when dopamine neurons are indiscriminately labeled.

In contrast, when we repeated these soma–axon recordings from isolated subtypes, we found highly similar signaling between striatal axons and SNc somas (Fig. 7c), resulting in high cross-correlations (Fig. 7d), and this was consistent across recordings (Fig. 7e). On average, the cross-correlation between soma and axon Δ*F*/*F* recordings was significantly higher compared to *DAT*^*+*^ recordings (Fig. 7e,f; mean = 0.65 for *Vglut2*^+^, 0.67 for *Calb1*^+^ and 0.58 for *Anxa1*^+^, compared to 0.37 for *DAT*; *P* values for comparison with *DAT*^*+*^ = 3 × 10^−4^ for *Vglut2*^+^, 0.003 for *Calb1*^+^ and 3 × 10^−4^ for *Anxa1*^+^, Mann–Whitney *U*-test with Bonferroni correction). Overall, we conclude that recording from isolated dopaminergic functional subtypes leads to highly similar signaling patterns between somas and axons in behaving mice.

## Discussion

In this study, we first found functional heterogeneity within the well-known *Aldh1a1*^*+*^ subtype (Extended Data Fig. 1d–i). This motivated our use of single-nucleus transcriptomics to refine the existing classification of dopamine neuron subtypes and led to the validation and characterization of a new subtype characterized by *Anxa1* expression within the previously described SNc *Aldh1a1*^*+*^ subtype (Fig. 1). We then isolated and recorded from this new *Anxa1*^*+*^ subtype, as well as the known *Calb1*^*+*^ and *Vglut2*^*+*^ subtypes, and found unique functional signaling patterns to rewards (Fig. 4d,g), aversive stimuli (Fig. 4f,g), accelerations (Fig. 2g) and decelerations (Fig. 2g). We made three main findings. (1) Although the *Calb1*^*+*^ and *VGlut2*^*+*^ subtypes show robust positive responses to unexpected rewards and aversive stimuli, such responses were not detected in the *Anxa1*^*+*^ subtype (Fig. 4g), even at striatal locations where its axons overlapped with the other reward-responsive subtypes (Fig. 4l). (2) Acceleration-correlated and deceleration-correlated responses were differentially observed in genetically distinct neurons, with *Anxa1* being acceleration correlated and *Vglut2* and *Calb1* being deceleration correlated (Fig. 2). (3) When dopaminergic subtypes were genetically separated, somatic transients correlated well with axonal transients (Fig. 7). These findings establish a connection between functional responses and genetic subtypes of dopamine neurons across a range of functional dimensions, validating the behavioral relevance of molecular classification schemes. Though here we found significant differences in functional responses between SNc dopamine neuron subtypes across different midbrain and striatal regions, fiber photometry records the mean fluorescence signal from populations of axons or cell bodies in the recording volume (a sphere ~300 µm in diameter). Thus, it is possible that some functional heterogeneity exists within the genetic subtypes at the single-cell/axon level, which should be explored in the future. In particular, the *Anxa1*^*+*^ subtype displayed a broader range of correlations between somas and axons compared to the other subtypes, and a few (<10%) of *Anxa1*^*+*^ recordings did display small increases in Δ*F*/*F* after reward (Fig. 4d, *Anxa1*^*+*^, bottom rows), mainly located more ventral in striatum (Fig. 4j and Extended Data Fig. 8j). Although this small fraction of recordings is close to the number expected by chance, another possible explanation is that the genetic strategy that we developed to access this subtype is not fully optimized, because *Anxa1* expression in SNc dopamine neurons is not binary. Instead, there is a gradient of expression, making it possible for weakly *Anxa1*-expressing neurons to express the reporter (Extended Data Fig. 5e–g), including a small GCaMP6f^+^ population that did not detectably express *Anxa1*. Any differences in function that correspond with differential *Anxa1* expression could explain the decreased axon–soma correlations in this subtype compared to *Vglut2* and *Calb1*. Furthermore, a small number of false positives (GCaMP6f^+^/Anxa1^−^ SNc cells) or GCaMP6f^+^/Anxa1^+^ VTA cells could also explain the small number of outlier reward responses observed in more ventral striatum. Although it is clear that the *Anxa1*^*+*^ subtype as labeled here is more functionally homogeneous than the previous populations studied in this region of SNc/striatum (that is, *Aldh1a1*^*+*^ and *DAT*), better genetic markers to access this subtype should continue to be explored. Regardless, similar signaling patterns to our reported averages for all subtypes have been observed in single-cell^9–11,14–18^ and single-axon^13^ recordings. This suggests that the functional differences that we observed between subtypes are due to the strong enrichment of particular functions at the single-cell level for specific subtypes, although confirmation will require functional recordings of the neurons within each subtype at the single-cell/axon level. Thus, genetic subtypes provide a tool to reproducibly access different dopamine neuron functions, which is particularly important given the literature’s many conflicting observations/hypotheses on the role of dopaminergic neurons.

Although the general assumption has been that all midbrain dopamine neurons robustly respond to unexpected rewards, there has been scattered evidence against this dogma. A few single-cell studies reported some SNc dopamine neurons that did not respond to rewards^14,45^, and axonal imaging recordings in dorsal striatum found several single axons not encoding rewards^13^. However, other studies found reward responses in similar regions^12,20,21^. Because we detected robust reward responses in *Calb1*^*+*^ and *Vglut2*^*+*^ neurons, but not in *Anxa1*^*+*^ neurons, and because these different subtypes have different midbrain distributions and striatal projection targets, our results may help explain the previous discrepancies; different subtype(s) may have been investigated based on the recording location in SNc or striatum. Furthermore, our functional characterization of *Vglut2*^*+*^ neurons agrees with previous recordings from overlapping soma/axon regions that reported aversive stimuli and reward signaling^9–11^, with insensitivity to reward size^10^. Based on these properties, such neurons have been proposed to signal novelty or salience^9,11^ or to reinforce avoidance of threatening stimuli^10^. Thus, of the three subtypes studied here that account for most SNc dopamine neurons, only the *Calb1*^*+*^ subtype displayed robust reward size sensitivity, a hallmark of RPE and value coding, suggesting a role in positive reinforcement learning^40^. Interestingly, the amplitude of the response to rewards and air puffs was larger than that to decelerations or accelerations, as seen in triggered averages, although this could be due, at least in part, to the greater imprecision in the identification of relevant accelerations/decelerations or a reduced probability of a transient at accelerations/decelerations compared to rewards/air puffs.

Previous research reported that many SNc dopamine neurons signal at accelerations during a variety of motor tasks but with differences in whether the neurons increase or decrease their firing at accelerations^13–18,29^. Because, in the present study, we found that such signaling patterns were differentially expressed by the different subtypes, and because their cell body and axon locations are anatomically biased, these previous discrepancies might also be explained by the unknowing recording of different subtype(s) across studies based on location. For example, recordings in more medial SNc/lateral VTA (*Calb1*^*+*^ location) found that most neurons decrease their firing at accelerations and respond to rewards^16^; recordings from dorsal striatum axons (*Anxa1*^*+*^ axon location) found increases in signaling at accelerations and no detectable reward responses^13^; and recordings from a broader range of locations (and, thus, subtypes) in SNc found neurons with both increases and decreases of firing at accelerations^14^—all of which agree with our results when considering subtype anatomical distributions.

Numerous hypotheses have been proposed to explain the function of fast dopamine signaling during locomotion: some suggest that they increase the probability of movement initiations or the vigor of movements^14,46^, whereas others propose that they function as a corollary discharge signal associated with particular actions and are involved in reward-based credit assignment^4^, motor learning^47,48^ or reward-independent reinforcement of particular movements^49^. Again, however, these differences in results and interpretations may lie in which dopamine neuron subtypes were recorded or manipulated in previous studies. For example, the initiation/vigor hypothesis is supported by the optogenetic activation of dorsal striatum axons^13^ (likely *Anxa1*^*+*^ axons), whereas the credit assignment hypothesis is supported by studies of medial SNc and lateral VTA neurons^4^ (likely *Calb1*^*+*^ somas). Future optogenetic perturbation studies focused on the specific subtypes described here should help to provide further understanding of their role in behavior. However, although research has shown that the pattern of dopamine release is consistent with the GCaMP transients reported here (at least in regions dominated by a particular subtype, such as the dorsal striatum^50^), any perturbation studies will also need to consider that many dopaminergic neurons co-release other neurotransmitters—*Vglut2*^*+*^ neurons co-release glutamate^51^, and *Aldh1a1*^*+*^ neurons may co-release GABA^52,53^ (although see ref. ^52^)—which likely play additional functional roles within striatum^51,54^.

When the diversity of dopaminergic neurons was taken into account, we found high correlation between somatic and axonal signaling. This is consistent with the classical view that striatal dopamine release is controlled by anterogradely propagating action potentials originating in midbrain somas rather than by local striatal modulation controlling dopamine release. This finding is also in agreement with previous reports demonstrating that cholinergic interneurons and dopamine axons in striatum are often de-synchronized during behavior^55^, making it difficult to explain the majority of dopamine release based on local cholinergic control. However, this does not exclude the possibility that local cholinergic modulation may still play a role in controlling dopamine release at specific behavioral timepoints. For example, striatal dopamine and acetylcholine signaling have been found to synchronize at certain times during behavior, such as at locomotion initiation or during turning^34,55^. Regardless, our results here provide evidence that axons track somatic signaling within dopaminergic subtypes, indicating that such subtypes should be considered to fully understand the mechanisms of dopamine release in striatum during behavior.

Finally, our results provide new potential research directions for different dopamine-related diseases, such as Parkinson’s disease, because there is emerging evidence that several of the subtypes studied here exist in humans^36^. The cell body locations and axonal projections of *Aldh1a1*^*+*^ match the pattern of dopamine loss in Parkinson’s disease^56,57^, and these neurons are especially vulnerable in Parkinson’s disease^35,36^, for which the *Aldh1a1*^+^ subtype has garnered considerable attention^35,36,47,58^. In contrast, *Calb1* and *Vglut2* neurons appear relatively spared^36,59^. Our identification and characterization of *Aldh1a1*^+^/*Anxa1*^+^, *Calb1*^*+*^ and *Vglut2*^*+*^ subtypes within the SNc, with markedly different responses to acceleration, deceleration, reward and aversive stimuli, warrants a reconsideration of the role of dopamine in motor and non-motor symptoms of Parkinson’s disease. For example, motor deficits may not be due to an absolute dopamine deficiency but, rather, to a loss of dopamine signaling from specific pro-motor subtypes such as *Anxa1*^*+*^ neurons^60^.

## Methods

### Experimental model and subject details

#### Animals

All experimental procedures were conducted in accordance with National Institutes of Health guidelines and were reviewed by the Northwestern Animal Care and Use Committee. Cre mouse lines were maintained heterozygous by breeding to wild-type C57BL6 mice. The Th-Flpo line and the Ai93D reporter line were maintained homozygous. The DAT-tTA mouse line was maintained heterozygous by breeding with the Ai93D reporter. The *Aldh1a1*-iCre and Anxa1-iCre lines were generated at Northwestern University by the Transgenic and Targeted Mutagenesis Laboratory. Mice were genotyped using primers detailed in Supplementary Table 1.

Both males and females were used for all experiments (for snRNA-seq experiments, three females and two males; for photometry recordings, in total seven males, 11 females, one unrecorded for *Vglut2*^+^; nine males, 11 females, one unrecorded for *Calb1*^+^; eight males, 12 females, one unrecorded for *Anxa1*^+^; 10 males, 14 females, two unrecorded for *Aldh1a1*^+^; and eight males, 13 females, six unrecorded for *DAT*^+^; see each section for sex numbers for each analysis). For indiscriminate labeling of SNc dopamine neurons, DAT-IRES-Cre mice (RRID: IMSR_JAX:027178) were injected with AAV1-CAG-FLEX-GCaMP6f virus (RRID: Addgene_100835). For labeling of SNc *Anxa1*^+^ neurons, Anxa1-iCre mice (new line) were injected with AAV1-CAG-FLEX-GCaMP6f virus (RRID: Addgene_100835). For labeling *Vglut2*^+^ or *Aldh1a1*^+^ dopamine neurons, we crossed Vglut2-IRES-Cre (RRID: IMSR_JAX:016963) or *Aldh1a1*-iCre mice (new line) with Th-2A-Flpo mice^28^, and offspring were injected with AAV8-EF1α-CreOn/FlpOn-GCaMP6f virus (RRID: Addgene_137122). For labeling *Calb1*^+^ dopamine neurons, we crossed Calb1-IRES2-Cre mice (RRID: IMSR_JAX:028532) with DAT-tTA (RRID: IMSR_JAX:027178), Ai93D (CreOn/tTAOn GCAMP6f reporter) (RRID: IMSR_JAX:024107) mice. This strategy labels some *Calb1*^+^ VTA dopamine neurons, as shown in Extended Data Fig. 1b, but these neurons can be avoided in recordings by restricting the optic fiber placement to striatum and not the nucleus accumbens, where VTA *Calb1*^+^ neurons project^28^. Still, to confirm that the observed signaling properties are not due to these labeled VTA neurons, we recorded from two Calb1-IRES2-Cre/Th-2A-Flpo mice injected with AAV8-EF1α-CreOn/FlpOn-GCaMP6f virus (same strategy as used for labeling the *Vglut2*^*+*^ and *Aldh1a1*^+^ subtypes above, which largely avoids VTA labeling). Recordings from these mice did not differ from those obtained from Calb1-IRES2-Cre/DAT-tTA/Ai93D mice (Figs. 2 and 4) and were, thus, included together in all analyses. In addition, to confirm that the different labeling strategy used for *Anxa1*^+^ did not affect the results, we recorded from four Anxa1-iCre/Th-2A-Flpo mice injected with AAV8-EF1α-CreOn/FlpOn-GCaMP6f (as used for the *Vglut2*^+^ and *Aldh1a1*^+^ subtypes above). As expected, recordings from these mice did not differ from those obtained from Anxa1-iCre mice injected with AAV1-CAG-FLEX-GCaMP6f virus (Figs. 2 and 4) and were, thus, included together in all analysis.

Adult mice were used for viral injections at 2–4 months of age.

### Method details

#### Generation and characterization of the *Aldh1a1*-iCre Line

Because our previous *Aldh1a1*-CreERT2 strain displayed substantial mosaicism, resulting in only weak GcaMP6f signals, we opted to generate an Aldh1a1-iCre strain (Extended Data Fig. 5a). The *Aldh1a1*-iCre line was generated at Northwestern University by the Transgenic and Targeted Mutagenesis Laboratory. In brief, a P2A peptide directly followed by iCre and a BGH polyA sequence were inserted after the last encoded amino acid of *Aldh1a1*, using CRISPR-mediated homology-directed repair (HDR) (guides 1–2; Supplementary Table 1). First, PRXB6/N embryonic stem cells were electroporated and screened for insertion and correct locus with multiple primer pairs (*Aldh1a1*-iCre insertion primers forward 1–3 and reverse 1–3; Supplementary Table 1), followed by Sanger sequencing of iCre^+^ clones from outside the homology arms through the construct to confirm fidelity of the insertion. Clone C7 was expanded and injected into blastocysts to generate chimeras and used for all experiments herein. *Aldh1a1*-iCre mice were genotyped using primer set 3 described above. To determine the expression fidelity of this allele, 0.4 µl of AAV5-EF1α-DIO-mCherry (RRID: Addgene_37083) was injected into SNc bilaterally (coordinates relative to bregma: *x* = ±1.45 mm, *y* = −3.15 mm, *z* = −3.1, −4.1, −4.4, −4.7 mm, 0.1 µl at each depth) in *n* = 4 adult mice. Three weeks later, mice were perfused, and brains were sectioned at 25 µm for immunofluorescence staining. Floating sections were first blocked for 24 h at 4 °C in PBS containing 0.03% Triton-X and 5% normal donkey serum. Sections were incubated with primary antibodies against *Aldh1a1* (goat, R&D Systems, AF5869, RRID:AB_2044597, 1:500 dilution), TH (mouse, Sigma-Aldrich, T2928, RRID:AB_477569; Pel-Freez Biologicals, P40101-0, RRID:AB_461064, 1:1,000 dilution) and mCherry (rat, Thermo Fisher Scientific, M11217, RRID:AB_2536611, 1:2,000 dilution) in blocking buffer for 24 h, followed by four washes in PBS-Tween 20 and incubation with secondary antibodies diluted 1:250 (donkey anti-goat Alexa Fluor 488 (Molecular Probes, A-11055, RRID:AB_2534102), donkey anti-mouse Alexa Fluor 647 (Thermo Fisher Scientific, A-31571, RRID:AB_162542), donkey anti-rabbit Alexa Fluor 647 (Thermo Fisher Scientific, A-31573, RRID:AB_2536183), donkey anti-rat Cy3 (Jackson ImmunoResearch, 712-165-153, RRID:AB_2340667) and DAPI (Thermo Fisher Scientific, 62248)) for 2 h at room temperature. Sections were then imaged at ×20 on an Olympus BX61VS slide scanner. For each brain, 4–5 sections spaced at least 100 µm apart and centered about the area of maximal viral recombination were counted for mCherry^+^/DAPI^+^/*Aldh1a1*^+^ and mCherry^+^/DAPI^+^/*Aldh1a1*^−^ cells (2,740 cells total) (Extended Data Fig. 5b,c). All images shown related to the validation or characterization of the *Aldh1a1*-iCre mouse line have been deposited in raw, unprocessed formats in Zenodo (10.5281/zenodo.7909331)^61^.

#### Generation and characterization of the Anxa1-iCre line

To access the *Anxa1*^*+*^ dopamine neurons, the Anxa1-iCre line (Extended Data Fig. 5d) was also generated by the Transgenic and Targeted Mutagenesis Laboratory, using similar methodologies as above. For CRISPR-mediated HDR, guides 3–4 (Supplementary Table 1) were used. Clones were screened for insertion using iCre genotyping primers (*Aldh1a1*-iCre insertion primers F3 and R3; Supplementary Table 1). To determine the expression fidelity of this allele, 0.4 µl of AAV5-EF1α-DIO-mCherry (RRID: Addgene_37083) was also injected into SNc bilaterally (*n* = 4 mice) at the same coordinates as above, and 25-µm sections were stained for immunofluorescence using the same protocol as above but with rabbit anti-Anxa1 antibody (Thermo Fisher Scientific, 71-3400, RRID: AB_2533983, 1:500 dilution) in place of *Aldh1a1*. Sections were then imaged at ×20 on an Olympus BX61VS slide scanner. Viral recombination occurred in cells with both high Anxa1 expression and faint Anxa1 expression (Extended Data Fig. 5g). To confirm that Anxa1-iCre recombination was limited to a subset within *Aldh1a1*-expressing dopamine neurons, we stained *n* = 2 Anxa1-iCre, TH-Flpo, RC::FrePe mice for *Aldh1a1* (1:500 dilution) and GFP (no antibody, endogenous fluorescence only—expression of which is dependent on both iCre and Flpo recombination) using the same protocol as above, which showed Aldh1a1 expression to be broader than Anxa1-iCre cumulative labeling, thus confirming that Anxa1-iCre expression was limited to a subset within Aldh1a1^+^ dopamine neurons and that our viral labeling results were not an artifact of insufficient viral delivery and/or diffusion (Extended Data Fig. 5h).

Because the Anxa1-iCre line appears to recombine reporters in both Anxa1-high and Anxa1-low cells, we performed an additional validation experiment to assess the overlap of Cre recombination with Anxa1 protein within the context of the GCaMP labeling experiments. We injected *n* = 4 Anxa1-iCre mice with the same AAV1-CAG-FLEX-GCaMP6f virus (RRID: Addgene_100835) used for GCaMP recordings in this line and co-stained with Anxa1 and *Aldh1a1* using the same protocol as above. Using this method, we confirmed that the vast majority of recombination occurs in either Anxa1-high or Anxa1-low cells (Extended Data Fig. 5e,f) and that 90% of these cells stain positive for *Aldh1a1* protein, corroborating the *Anxa1*^*+*^ subtype as a subset of *Aldh1a1*-expressing dopamine neurons. All images shown related to the validation or characterization of the Anxa1-iCre mouse line have been deposited in raw, unprocessed formats in Zenodo (10.5281/zenodo.7909331)^61^.

#### snRNA-seq

To isolate nuclei for snRNA-seq library generation, *n* = 5 DAT-IRES-CRE (RRID:IMSR_JAX:027178), CAG-Sun1/sfGFP (RRID:IMSR_JAX:021039) mice (three females, two males) were killed and rapidly decapitated for extraction of brain tissue. This cross results in specific labeling of DAT-expressing (that is, dopaminergic) nuclei with a nuclear membrane protein fused to GFP, allowing isolation of these nuclei by fluorescence-activated cell sorting (FACS). A 2–3-mm-thick block of ventral midbrain tissue was dissected out and collected for subsequent isolation. Tissue was dounce homogenized in a nuclear extraction buffer (10 mM Tris, 146 mM NaCl, 1 mM CaCl_2_, 21 mM MgCl_2_, 0.1% NP-40, 40 U ml^−1^ Protector RNAse inhibitor (Roche, 3335399001)). Dounce homogenizer was washed with 4 ml of a washing buffer (10 mM Tris, 146 mM NaCl, 1 mM CaCl_2_, 21 mM MgCl_2_, 0.01% BSA, 40 U ml^−1^ Protector RNAse inhibitor) and filtered through a 30-µm cell strainer. After three rounds of washing by centrifugation (500*g* for 5 min) and resuspension in a nuclei resuspension buffer (10 mM Tris, 146 mM NaCl, 1 mM CaCl_2_, 21 mM MgCl_2_, 2% BSA, 0.02% Tween 20), nuclei suspension was stained with DAPI and filtered through a 20-µm strainer. This nuclei suspension was then sorted via FACS with a 100-µm nozzle at a frequency of 30,000 and pressure of 20 psi, with gates set for isolation of GFP^+^ singlet nuclei (Extended Data Fig. 3a). A total of 50,500 nuclei were sorted across all samples, which was subsequently used for preparation of two 10x Genomics Chromium libraries (one for pooled male mice, one for pooled female mice).

Library preparation was performed by the Northwestern University NUSeq Core Facility. Nuclei number and viability were first analyzed using Nexcelom Cellometer Auto 2000 with AOPI fluorescent staining method. In total, 16,000 nuclei were loaded into the Chromium Controller (10x Genomics, PN-120223) on a Chromium Next GEM Chip G (10x Genomics, PN-1000120) and processed to generate single-cell gel beads in the emulsion (GEM) according to the manufacturer’s protocol. The cDNA and library were generated using Chromium Next GEM Single Cell 3′ Reagent Kits v3.1 (10x Genomics, PN-1000286) and Dual Index Kit TT Set A (10x Genomics, PN-1000215) according to the manufacturer’s manual with the following modification: PCR cycle used for cDNA generation was 16, and the resulting PCR products were size-selected using 0.8× SPRI beads instead of 0.6× SPRI beads as stated in the protocol. Quality control for the constructed library was performed by Agilent Bioanalyzer High Sensitivity DNA Kit (Agilent Technologies, 5067-4626) and Qubit DNA HS Assay Kit for qualitative and quantitative analysis, respectively.

The multiplexed libraries were pooled and sequenced on Illumina NovaSeq 6000 sequencer with paired-end 50 kits using the following read length: 28 bp Read1 for cell barcode and unique molecular identifier (UMI) and 91 bp Read2 for transcript. Raw sequence reads were then demultiplexed, and transcript reads were aligned to mm10 genome using CellRanger with –include-introns function.

#### Stereotaxic viral injections for fiber photometry experiments

Adult mice (postnatal 2–4 months old) were anesthetized with isoflurane (1–2%), and a 0.5–1-mm-diameter craniotomy was made over the right substantia nigra (−3.25 mm caudal, +1.55 mm lateral from bregma). A small volume (0.4 μl total) of virus (AAV8-EF1α-CreOn/FlpOn-GCaMP6f (RRID:Addgene_137122, titer 6.10 × 10^13^) for *Aldh1a1*-iCre/Th-Flpo, Vglut2-IRES-Cre/Th-Flpo and Calb1-IRES2-Cre/Th-Flpo mice or AAV1-CAG-FLEX-GCaMP6f (RRID:Addgene_100835, titer 2.00 × 10^13^) for DAT-Cre or Anxa1-iCre mice), diluted 1:1 in PBS, was pressure injected through a pulled glass micropipette into the SNc at four depths (−3.8, −4.1, −4.4 and −4.7 mm ventral from dura surface, 0.1 μl per depth). After the injections, the skull and craniotomy were sealed with Metabond (Parkell), and a custom metal headplate was installed for head fixation. The location of recording sites was marked on the surface of the Metabond for future access. For Calb1-IRES2-Cre/DAT-tTA/Ai93D mice, which express GCaMP6f endogenously, no injection was conducted, and only the headplate was implanted at this time. Four weeks were allowed for GCaMP6f expression to ramp up and fill dopaminergic somas in SNc and axons in striatum. For more details, see the online protocol (10.17504/protocols.io.5qpvor8zxv4o/v1)^62^.

#### Training and behavior

Starting 1–2 weeks after injection, mice were head-fixed with their limbs resting on a one-dimensional cylindrical Styrofoam treadmill ~20 cm in diameter by 13 cm wide in the dark. Mice were habituated on the treadmill for 3–10 d until they ran freely and spontaneously transitioned between resting and running. Rotational velocity of the treadmill during locomotion was sampled at 1,000 Hz by a rotary encoder (E2-5000, US Digital) attached to the axle of the treadmill and a custom LabView program.

After mice ran freely, a subset of mice was water restricted and received unexpected water rewards, aversive air puffs and light stimuli while on the treadmill, using a custom LabView program. Large-volume (16 μl) and small-volume (4 μl) water rewards were delivered through a waterspout gated electronically through a solenoid valve, which was accompanied by a short ‘click’ noise. Air puffs were delivered by a small spout pointed at their left whiskers, which was connected to a ~20-psi compressed air source and triggered electronically through the opening of a solenoid valve for 0.2 s. Triggering of this solenoid was also accompanied by a ‘click’ noise. For light stimuli, a blue LED placed ~30 cm in front of the head-fixed mouse was electronically triggered for 0.2 s. Rewards, air puffs and light stimuli were alternated at random during recordings and delivered at pseudo-random time intervals (10–30 s between any two stimuli).

For more details, see the online protocol (10.17504/protocols.io.4r3l27yj4g1y/v1)^63^.

#### Fiber photometry

Four weeks after injection, mice were once again anesthetized, and a small craniotomy (1 mm in diameter) was drilled through the Metabond and skull, leaving the dura and cortex intact. Craniotomies were made at different locations depending on the experiment, which were pre-marked during the injection surgery: for SNc −3.25 mm caudal, +1.55 mm lateral from bregma and different locations over striatum (for example, −1.1 mm caudal, +2.8 mm lateral or +0.5 mm caudal, +1.8 mm lateral). The craniotomies were then sealed with Kwik-Sil (World Precision Instruments).

After the mice recovered from this short (10–15 min) surgery for 1 d, they were head-fixed on the linear treadmill, and the Kwik-Sil covering the craniotomies was removed. One or two optical fibers (200-μm diameter, 0.57 NA, Doric MFP_200/230/900-0.57_1.5m_FC-FLT_LAF) were lowered slowly (5 μm s^−1^) using a micromanipulator (Sutter Instrument, MP285) into the brain to various depths measured from the dura surface. In the striatum, recording depths ranged from 1.6 mm to 4.1 mm; in SNc, depths ranged from 3.5 mm to 4.5 mm. Recordings started at 1.6 mm in striatum and 3.5 mm in SNc, but if no Δ*F*/*F* transients were detected at those depths, the fiber was moved down in increments of 0.25–0.5 mm in striatum or 0.15–0.2 mm in SNc, until transients were detected. From there, a 15-min recording was obtained, and the fiber was moved further down in the same increments. Subsequent recordings were obtained until a depth was reached where transients were no longer detected, at which point the fiber was pulled out of the brain slowly (5 μm s^−1^).

A custom-made photometry setup was used for recording. Blue excitation light (470-nm LED, Thorlabs, M70F3) and purple excitation light (for the isosbestic control) (405-nm LED, Thorlabs, M405FP1) were coupled into the optic fiber such that a power of 0.75 mW emanated from the fiber tip. Then, 470-nm and 405-nm excitation were alternated at 100 Hz using a waveform generator, each filtered with a corresponding filter (Semrock, FF01-406/15-25 and Semrock, FF02-472/30-25) and combined with a dichroic mirror (Chroma Technology, T425lpxr). Green fluorescence was separated from the excitation light by a dichroic mirror (Chroma Technology, T505lpxr) and further filtered (Semrock, FF01-540/50-25) before collection using a GaAsP PMT (H10770PA-40, Hamamatsu; signal amplified using Stanford Research Systems SR570 preamplifier). A PicoScope data acquisition system was used to record and synchronize fluorescence and treadmill velocity at a sampling rate of 4 kHz.

For more details, see the online protocol (10.17504/protocols.io.4r3l27yj4g1y/v1)^63^.

#### Histology

Immediately after the last recording, mice were perfused transcardially with PBS (Thermo Fisher Scientific) and then 4% paraformaldehyde (PFA) (Electron Microscopy Sciences). Brains were stored in PFA at 4 °C overnight and then transferred to 40% sucrose (Sigma-Aldrich) for at least 2 d before sectioning. Coronal slices (50 μm thick) were cut on a freezing microtome and stored at 4 °C in PBS. For immunostaining of dopaminergic neurons, sections were washed in PBS, blocked in PBS + 0.3% Triton-X (Sigma-Aldrich) + 5% normal donkey serum (Sigma-Aldrich), incubated overnight with primary antibodies sheep anti-tyrosine hydroxylase (1:1,000 dilution, RRID:AB_461070) and rabbit anti-GFP, which recognizes GCaMP6f (1:1,000 dilution, RRID:AB_221569), washed again in PBS + 0.3% Triton-X and then incubated with secondary antibodies tagging tyrosine hydroxylase with Alexa Fluor 555 (donkey anti-sheep Alexa Fluor 555, RRID:AB_2535857) and GCaMP6f with Alexa Fluor 488 (donkey anti-rabbit Alexa Fluor 488, RRID:AB_2313584). Images of SNc and striatum were acquired on an Olympus or Keyence slide scanner (VS120 or BZ-X810, respectively) for verification of injection accuracy and fiber placement. Other brains were mounted and imaged without immunostaining for fiber placement. Histology is not available for two of 14 DAT^+^ mice and one of five Calb1^+^ mice, and fiber tracks could not be identified for two of 16 *Vglut2*^*+*^ mice.

### Quantification and statistical analysis

Data were analyzed using custom code in MATLAB and R (in RStudio). This code is available at GitHub and has been deposited in Zenodo (10.5281/zenodo.7900531)^64^.

### Statistics and reproducibility

Some fiber photometry recordings were excluded from analysis based on exclusion criteria that were fixed for all subtypes and which were determined independently of subsequent analysis. More details on these exclusion criteria can be found in the subsection titled ‘Criteria for recording inclusion’ below and fall mainly into two categories: signal-to-noise (some recordings had low signal-to-noise ratios and were, thus, excluded) or behavioral criteria (for example, recordings were mice were not running were not included in locomotion analysis, and recordings where mice did not lick to the delivered rewards were not included in reward analysis).

For tests of statistical significance, all *P* values reported were calculated using non-parametric (not relying on assumptions that the data are drawn from any particular distribution) two-sided tests: Wilcoxon signed-rank tests for one-sample tests, Mann–Whitney *U*-tests (also known as Wilcoxon rank-sum test) for two-sample unpaired tests and paired Wilcoxon signed-rank tests for two-sample paired tests. The specific test used is stated in the main text and figure legends where *P* values are reported and in each methods subsection. All tests were corrected for multiple comparisons using the Bonferroni correction: multiplying the *P* values by the number of comparisons made. This correction sometimes resulted in *P* values above 1, but these were reported as 1. Stars (*) for reporting *P* values in figures were used in following convention: ****P < 0.0001, ***P < 0.001, **P < 0.01, *P < 0.05 (NS) and *P* ≥ 0.05. Statistical calculations of RNA-seq data in Seurat use a Wilcoxon rank-sum test, and Bonferroni corrections in these cases are based on the number of all genes in the dataset rather than only the number of genes being tested, as tested genes are likely to be non-randomly selected.

No statistical methods were used to pre-determine sample sizes, but our sample sizes are similar to those reported in previous publications (refs. ^13,14,16^). The experiments were not randomized. Data collection and analysis were not performed blinded to the conditions of the experiments, as different subtypes require recording from different (although partially overlapping) regions of striatum, due to their different projection regions. Exclusion criteria (signal-to-noise and behavioral) for recordings, however, were selected blind to subtype identity and subsequent analysis.

For reproducibility, the new Anxa1^+^ subtype was identified from analysis of both a meta-dataset of existing scRNA-seq (Extended Data Fig. 2) and a new dataset of snRNA-seq (Fig. 1). Subtype marker expression was corroborated using the Allen Brain Atlas in situ hybridization dataset (Extended Data Fig. 4). Locomotion signaling was corroborated using several complementary analyses (cross-correlation with acceleration and different triggered averages (Fig. 2f–h)). PCA analysis and reward/air puff signaling were corroborated using different normalization settings (Extended Data Figs. 7a,b and 8g). Functional analysis of different subtypes was corroborated in recordings from striatum and SNc (Extended Data Fig. 9).

### Integration of snRNA-seq datasets

Data from four previous single-cell studies (refs. ^24–27^; see Supplementary Table 1 for data sources) were acquired for integration using Seurat version 3.2.0. For Saunders et al.^25^ data, specific clusters identified as TH^+^ substantia nigra neurons (SN clusters 4-1, 4-2, 4-3, 4-4, 4-5, 4-6, 4-7, 4-8, 4-9 and 3-7) were subsetted and used integration. Violin plots of number of reads and number of genes for each dataset were generated and used to determine cutoffs for pre-filtering of each dataset before integration to remove doublets or low-quality cells (Extended Data Fig. 2c).The following filters were ultimately applied: Saunders et al.: nFeatures <3,500, mitochondrial read % <25, nCount <10,000; Tiklova et al.: nFeatures >6,000, mitochondrial read % <10, nCount <3,500,000; and Kramer et al.: nFeatures >5,000, nCount >400,000. Datasets were normalized individually and integrated using the recently described SCTransform pipeline^65^ with default settings and regression on percent mitochondrial reads. PCA was performed on the subsequent integrated dataset, and an elbow plot was used to determine the number of PCs used for clusters (18 PCs were ultimately used). Clustering was performed using the standard Seurat pipeline at default settings, resulting in eight clusters (Extended Data Fig. 2a). Determination of marker genes for clusters was performed using the FindAllMarkers command in Seurat on the RNA assay with the following settings: min.diff.pct = 0.20, only.pos = TRUE, min.pct = 0.05. To explore any potential inclusion of a unique group of cells stemming from only a single dataset, we re-clustered our dataset using the LIGER R package version 2.0.1, which differs from Seurat dataset integration in that it is designed to account for the potential inclusion of unique cell types stemming from only individual samples being integrated^66^. Clustering with LIGER revealed a cluster of distantly related cells that came entirely from Tiklova et al.^26^ (Extended Data Fig. 2d). Due to the distinct signature of these cells that did not match the clusters they were placed in using the Seurat integration, these cells were subsequently filtered out of our dataset. After this, all clusters were represented by all source datasets (Extended Data Fig. 2e). Violin plots of the top two defining markers per cluster were generated using the Seurat VlnPlot command with default settings (Extended Data Fig. 2f).

### Analysis of snRNA-seq data

Outputs from CellRanger were read into Seurat version 4.0.2 using the Read10X command for each sample. Numbers of UMIs, features and mitochondrial reads were plotted for each dataset (Extended Data Fig. 3c) and used to determine cutoffs for quality control pre-filtering of each sample; nuclei with fewer than 500 unique features were removed from each dataset. The male and female datasets were then normalized and integrated using the SCTransform V2 pipeline^67^ using all default settings and regression on percent mitochondrial reads. In total, the integration resulted in a final dataset of 12,065 nuclei, with a mean UMI count of 3,435 and a mean of 1,683 features. Clustering was performed using the Seurat FindClusters command using 30 PCs and a resolution of 0.5. Differential expression tested was performed using the FindAllMarkers command on the SCT assay with default settings, with the exception of logfc.threshold = 0.15 to better detect differential expression of genes with low overall detection rates in the dataset. Determination of *Sox6*^+^ and *Calb1*^+^ significant clusters was made using a Wilcoxon rank-sum test by running the FindAllMarkers Seurat command with the following settings: features = c(‘Sox6’, ‘Calb1’), min.pct = 0, min.diff.pct = 0, logfc.threshold = 0 and only.pos = TRUE.

To identify clusters with weak dopaminergic characteristics, we used the Seurat FindMarkers() command to test for differential expression of *TH*, *DAT* and *DDC* in all clusters to determine which significantly underexpressed this combination of classic dopamine neuron markers (Wilcoxon rank-sum test, Bonferroni-corrected *P* < 0.05), resulting in clusters 12, 14 and 15 being labeled as neurons with weak dopamine characteristics. Cluster 8 also satisfied these requirements but had significant expression for *Gad2* and *Crhbp* and, thus, likely represents a previously described dopamine neuron subtype in the VTA^28^. Cluster 13 was also excluded, as it was determined to likely be doublets of dopamine neurons and oligodendrocytes based on significant expression of Mbp (Wilcoxon rank-sum test, Bonferroni-corrected *P* < 0.05), which was not present in any other clusters.

To better visualize the expression of marker genes, we then performed zero-preserving zero-imputation using ALRA^68^ via the SeuratWrappers R package version 0.2.0, which aims to increase the detection of poorly detected genes while preserving true biological zeros. Zero-imputed data were used solely for visualizations of features, as seen in Fig. 1b,c and Extended Data Fig. 3e,h, but not for any statistical determination of differential expression. Heat map of the top four marker genes for each cluster (Extended Data Fig. 3g) was generated using the top four differentially expressed genes (DEGs) (determined per average log fold change) for each cluster, filtering for only unique genes. Clusters were identified using known marker genes that were differentially expressed as well as by mapping the expression of novel DEGs identified either using the Seurat FindAllMarkers() command with or without zero imputation or by examining outliers of scatter plots that directly compared the average gene expression for all genes between two individual clusters (Extended Data Fig. 4c), followed by using the Allen Mouse Brain Atlas in situ hybridization dataset^69^ (Extended Data Fig. 4d,e). Cluster 9 was inferred to be the *Vglut2*^*+*^ subtype investigated based on the following gene signature, *Vglut2*^+^*/Otx2*^−^*/Sox6*^−^*/Aldh1a1*^−^*/Calb1*^+^*/Crhbp*^−^, and was further distinguished from another similar cluster (cluster 10) based on post-zero-imputed expression of markers, including *Tmem163* and *Gsta4*. Cluster 11 was inferred to be the *Calb1*^+^ subtype investigated based on the following gene signature: *Calb1*^+^*/Vglut2*^−^*/Otx2*^−^*/Sox6*^−^*/Aldh1a1*^−^.

A dendogram of hierarchical clustering for Extended Data Fig. 3f was produced using the Seurat function BuildClusterTree(). Cluster numbers were assigned based on the order of their branching from the resulting dendrogram. Because the height of branch points between clusters provides an approximation of the relatedness of said clusters, but without any associated statistics, we developed a custom R script for determining if one pair of clusters (pair A) is more closely related than another pair of clusters (pair B) by calculating the correlation coefficient of the average gene expression values for all genes for each pair, respectively, and then bootstrapping a 95% confidence interval for the difference in correlation coefficients between pair A and pair B by repeated random sampling with replacement from the gene lists, calculating new correlation coefficients for each pair and calculating the difference in correlations for each repetition. This was repeated 1,000 times. A pair of clusters that has a greater correlation coefficient and a 95% confidence interval for the difference in correlation coefficients that does not overlap with zero was, thus, deemed significantly closer transcriptionally related. For example, performing this analysis to compare the *Anxa1*^+^*/Aldh1a1*^*+*^ and *Anxa1*^−^*/Aldh1a1*^*+*^ cluster pair to the putative *Vglut2*^*+*^ and *Calb1*^*+*^ cluster pair shows that the former pair is more closely related than the second.

### Cluster stability and homogeneity analyses

To quantify how homogenous each cluster is and to uncover potential further subdivisions that may exist within our clusters, we applied two approaches. First, we generated a measure of stability for each cluster through random downsampling and reclustering of the dataset using custom R scripts. In brief, the full dataset was downsampled to 80% of the nuclei at random and re-run through the Seurat analysis pipeline (including PCA, uniform manifold approximation and projection (UMAP) embedding and clustering). After clustering, each new cluster was compared to all the original clusters through a Jaccard similarity index, and the maximally similar value was stored. These values were normalized by dividing by 0.8, the theoretical maximum Jaccard index that could be achieved. This process was repeated 100 times and graphed (Extended Data Fig. 4a). By comparing the similarity of the new clusters to the old clusters, we could assess how stably cells co-clustered when information was missing, based on the same principles used in the scclusteval R package for determining optimal clustering parameters.

To understand potential sources of lower cluster stability values (for example, two clusters being grouped together as one or additional small clusters being divided into two adjacent clusters), we performed clustering in Seurat using the FindClusters() command at iteratively higher cluster resolutions and mapped the development of new clusters relative to the previous set (Extended Data Fig. 4b).

### Photometry data pre-processing

Simultaneous traces (velocity from rotary encoder, trigger signals for reward, air puff and light stimuli delivery, licking from a lick sensor, fluorescence detected by photomultiplier tubes (PMTs) from one or two optic fibers and output from waveform generator used to alternate 405-nm and 470-nm illumination every 10 ms) were collected at 4 kHz by a PicoScope 6 data acquisition system. Fluorescence collected during 405-nm or 470-nm illumination (20 time bins for each pulse of 405-nm or 470-nm excitation) was separated using the binary output from the waveform generator. For each transition period between illumination sources, five time bins were excluded to remove transition times. Traces were then re-binned to 100 Hz by averaging every 40 time bins for velocity and every 40 time bins for 405-nm and 470-nm fluorescence traces (but including only 15 of 40 bins for each source: excluding 20 bins when the alternate source was on and five transition bins).

We first corrected fluorescence traces for background signal (intrinsic fluorescence and any illumination bleed-through) by subtracting 85% of the baseline (baseline defined as 8th percentile over a 20-s window). This 85% was estimated from photometry recordings from cortex, which was unlabeled (no GCaMP expression), obtained from 10 recordings from five mice. The 405-nm and 470-nm fluorescence traces were corrected independently. To calculate Δ*F*/*F*, traces were then normalized by baseline fluorescence division (8th percentile over a 20-s window) separately for 405 nm and 470 nm. The subtraction and normalization steps together corrected for bleaching and removed any slow drifts in baseline. Next, traces were converted to Δ*F*/*F* units (baseline at 0) by subtracting the baseline (median of all non-transient bins for 470-nm traces and median of all bins for 405-nm traces).

For comparison of traces between dopaminergic subtypes, Δ*F*/*F* traces were normalized so that the baseline remained at 0 and the largest transient peak for each trace was 100%. Throughout all figures, normalized Δ*F*/*F* units refer to this normalization (0–100 scale). The 405-nm traces were normalized using the amplitude of the largest peak from the corresponding 470-nm traces. Example raw traces in Figs. 2c, 4c and 7c and Extended Data Figs. 1d and 10a show non-normalized traces.

Raw data^70^ and pre-processed data^71^ (Δ*F*/*F*) have been uploaded to Zenodo (10.5281/zenodo.7871634 and 10.5281/zenodo.7871982, respectively).

### Criteria for recording inclusion

Exclusion criteria were identical for all subtypes and were determined independently of subtype identity and subsequent analysis.

Only recordings with signal-to-noise ratios greater than 10 were included in the analysis. To calculate signal-to-noise ratios for each recording, we selected well-isolated transients, as defined by having a large, fast rise (30 Δ*F*/*F* s^−1^) immediately followed by a decay. We first removed all slow fluctuations except transients in (non-normalized) Δ*F*/*F* traces by subtracting the 8th percentile over a window 2–3 times the width of observed Δ*F*/*F* transients (250 bins, 2.5 s) and then smoothed the resulting trace over a 0.2-s window (20 bins) to reduce noise. Transient rises and decays were identified by locating the zero-crossings on the derivative of the trace, also smoothed over a 0.2-s window. Only clearly isolated transients were included—those with a rise greater than 30 Δ*F*/*F* s^−1^ followed by a decay greater than −5 Δ*F*/*F* s^−1^. Traces with fewer than 0.2 transients per second were excluded. Signal values for each recording were calculated as the 80th percentile of isolated transient peaks. Noise for each recording was calculated by smoothing each (non-normalized) Δ*F*/*F* trace over 10 bins (0.1 s) and then subtracting this smoothed trace from the original Δ*F*/*F* trace and using the s.d. of the resulting trace as the noise value. The signal and noise values were divided to obtain signal-to-noise for each trace. These steps for determining signaling to noise for each trace were not used for any further analysis.

Δ*F*/*F* traces from 405-nm illumination (isosbestic control) were used to remove any movement artifacts. Although GCaMP6f fluorescence intensity is dependent on calcium concentration when excited with 470-nm light, it is still fluorescent but in a calcium-independent way when excited with 405-nm light^30^. Therefore, calcium transients in neurons are detected with 470-nm illumination but are absent with 405-nm illumination, whereas movement artifacts are present in both traces. Movement artifacts were identified using the 405-nm traces from each recording as follows. Non-normalized 405-nm Δ*F*/*F* traces were smoothed over a 10-bin window (0.1 s). This smoothed trace was subtracted from the original 405-nm Δ*F*/*F* trace, so that only the noise remained (same process as used above for 470-nm traces to separate noise and signal). A maximum noise value was calculated as the maximum absolute value of this noise trace. Any bins in the original 405-nm Δ*F*/*F* trace more than three times this maximum noise (or three times below the maximum noise) were excluded from further analysis. Additionally, any sequential bins that were above maximum noise (or below maximum noise) for longer than 0.2 s (20 bins, less than half the width of observed calcium transients) were also excluded, with an additional 0.1 s (10 bins) on both sides also excluded. Any bins removed from the 405-nm Δ*F*/*F* trace were also removed in the corresponding 470-nm Δ*F*/*F* and velocity traces. If more than 5% of the bins in a recording met these movement artifact exclusion criteria, the entire recording was excluded.

Behavioral criteria were also used to determine the inclusion of recordings for each type of analysis. For details on these criteria, refer to each corresponding subsection below. In some recordings, mice were running on the wheel but did not receive rewards or air puffs, and, thus, these recordings are included in the locomotion analysis in Fig. 2 but not in Fig. 4. On other recordings, mice were receiving rewards and air puffs but did not run, and, thus, they were included in Fig. 4 but not in Fig. 2. Additionally, in a few recordings, mice were receiving air puffs and rewards but were not licking to consume the reward (possibly due to satiety), and, thus, these recordings were used for air puff analysis but not rewards (1/29 for *Vglut2*^+^, 0/17 for *Calb1*^+^ and 6/57 for *Anxa1*^+^). The subset of recordings where mice were running, consuming rewards and receiving air puffs is included in Fig. 6 (*n* = 16 for *Vglut2*^+^, *n* = 11 for *Calb1*^+^ and *n* = 16 for *Anxa1*^+^), but this was not the case for all recordings, and these others were used for analysis of only the behavioral variables that were relevant.

### Analysis of signaling during locomotion

Recordings included for locomotion analysis come from 12 *Vglut2*^*+*^ mice (four males, seven females, one unrecorded), six *Calb1*^*+*^ mice (five males, one femle), nine *Anxa1*^*+*^ mice (four males, five females), 14 *Aldh1a1*^*+*^ mice (five males, eight females, one unrecorded) and 14 *DAT*^*+*^ mice (three males, seven females, two unrecorded).

All times within a 5-s window after any stimulus was delivered (reward, air puff) were excluded from all locomotion analysis described below; this excluded reward/air puff movement reactions and consumptive licking behavior from the locomotion analysis.

Only locomotion time bins were included for locomotion analysis in Fig. 2 and Extended Data Figs. 1, 6, 7 and 9. Locomotion versus rest bins were selected using a double threshold on the velocity trace in both positive and negative directions (thresh1 = ±0.024 m s^−1^ and thresh2 = ±0.010 m s^−1^). Isolated one-bin-long locomotion periods (no other movement within two bins on either side) were excluded as well as rest periods shorter than 0.5 s. Time bins were considered as locomotion periods only if they lasted longer than 0.5 s and had an average velocity greater than 0.2 m s^−1^. For a recording to be included in the locomotion analysis, the recording needed to include a total of at least 100 s of locomotion.

Acceleration was calculated from the velocity traces as the difference between consecutive treadmill velocity time bins (first smoothed over six bins, 0.06 s) and then multiplied by the sampling frequency (100 Hz) for proper m s^−^^2^ units.

Cross-correlations between Δ*F*/*F* and acceleration (Figs. 2d,f and 3a and Extended Data Figs. 1e,f, 6c,h,i and 9h,g) were calculated for locomotion periods only (defined above) using MATLAB’s crosscorr function over a 1-s lag window (100 time bins). The same process was used to calculate the cross-correlation between corresponding 405-nm Δ*F*/*F* traces and acceleration, and any recording with a peak cross-correlation (between 405-nm Δ*F*/*F* trace and acceleration) above 0.1 was excluded from all locomotion analysis. This same strategy was also used to calculate cross-correlations between different variables (licking versus velocity, licking versus acceleration, licking versus Δ*F*/*F* and velocity versus Δ*F*/*F*) as shown in Extended Data Fig. 7h. Averages per mouse (Extended Data Fig. 6i) were obtained by averaging together the cross-correlations for all recordings made from the same mouse.

For triggered averages of Δ*F*/*F* on accelerations and decelerations (Figs. 2g and 5a and Extended Data Figs. 6b, 7d and 9j), isolated large accelerations and decelerations were selected by first locating the zero-crossings on the acceleration trace (points where the acceleration trace crosses zero, going from negative to positive or vice versa), considering individual accelerations/decelerations the interval between two zero-crossings of the trace. Accelerations/decelerations were included if they had a duration of at least 50 ms (0.05 s) and a peak greater than 2 m s^−^^2^ (accelerations) or lower than −2 m s^−^^2^ (decelerations), but only if they were not surrounded by other large accelerations or decelerations (no acceleration >2 m s^−^^2^ or <−2 m s^−^^2^ in a window of 0.25 s on either side). Triggered averages were the result of averaging 135 ± 77 accelerations and 138 ± 101 decelerations (mean ± s.d.) per recording (Fig. 2g). For an example recording of each subtype showing that recording’s triggered average with a heat map of each individual event that contributes to the recording’s average, see Extended Data Fig. 7d. The probability that Δ*F*/*F* transients follow accelerations is 57.5% for *Anxa1*, and the probability that transients follow decelerations is 62.4% for *Vglut2*^+^ and 62.3% for *Calb1*^+^, as shown in Extended Data Fig. 7f. This was obtained by first calculating for each event the integral of the Δ*F*/*F* trace within a 0.75-s window from the start of the acceleration/deceleration (*t* = 0 s), after subtracting the Δ*F*/*F* value at *t* = 0 s. Histograms were then obtained for the percent of accelerations/decelerations per recording with positive values for this calculation.

Conversely, for triggered averages of acceleration on Δ*F*/*F* transient peaks (Fig. 2h and Extended Data Figs. 6d, 7e and 9i), we selected well-isolated transients from non-normalized Δ*F*/*F* traces, as defined by having a large, fast rise (30 Δ*F*/*F* s^−1^) immediately followed by a decay (as used in the calculation of signal-to-noise ratio above). Triggered averages were the result of averaging 423 ± 271 transients (mean ± s.d.) per recording (Fig. 2h). For an example recording of each subtype showing that recording’s triggered average with a heat map of each individual event that contributes to the recording’s average, see Extended Data Fig. 7e.

For triggered averages on movement onsets and offsets (Extended Data Fig. 7g), we started with the transitions between locomotion and rest bins as selected above. Often mice moved backwards or jittered before starting to run or after stopping, so to select only clean onsets we only included transitions that reached a velocity of 0.4 m s^−1^ within 0.75 s of starting to move, with an initial acceleration peak of at least 1 m s^−^^2^ and with no negative velocities below –0.05 m s^−1^ before this strong acceleration. For offsets, the symmetric conditions were required (stopping from a velocity of at least 0.4 m s^−1^ within 0.75 s, with a final deceleration of at least –1 m s^−^^2^ and no negative velocities below –0.05 m s^−1^ at the end of the offset). For plotting of cross-correlation and triggered averages above, traces were smoothed over five time-lag bins (0.05 s). Shaded areas represent the mean ± s.e.m. across recordings, and accompanying heat maps show cross-correlations/triggered averages for all individual recordings. Heat maps in Extended Data Fig. 1f were sorted by the integral of the Δ*F*/*F*-acceleration cross-correlation at positive lags (see below), whereas heat maps in Fig. 2f–h and Extended Data Figs. 6b–d and 7g–i were sorted by PC1/PC2 angle (see PCA subsection below)—other than that, the data plotted in Extended Data Fig. 1f and in Fig. 2f and Extended Data Fig. 6c are the same (with the addition of *Anxa1*^+^).

SNc recordings (Extended Data Fig. 9h–k) were analyzed in the same manner as striatal recordings. Recordings included for locomotion analysis in SNc come from 11 *Vglut2*^*+*^ mice (five males, six females), three *Calb1*^*+*^ mice (three males), eight *Anxa1*^*+*^ mice (four males, four females), 13 *Aldh1a1*^*+*^ mice (five males, seven females, one unrecorded) and eight *DAT*^*+*^ mice (two males, three females, two unrecorded).

For the initial functional characterization shown in Extended Data Fig. 1h,i, differences in locomotion signaling were quantified by calculating the integral of the cross-correlation between Δ*F*/*F* and acceleration at positive lags (0–1 s), where positive values indicate a peak in the cross-correlation and, thus, Δ*F*/*F* transients after accelerations, whereas negative values indicate a trough and, thus, Δ*F*/*F* transients after decelerations. For the quantification of acceleration/deceleration signaling across depths in striatum shown in Extended Data Fig. 1h, depth from surface was defined as the depth at which the fiber tip was located from the brain surface, as measured by the micromanipulator used to move the fiber during photometry. To reduce overlap between data points at the same depth plotted, a random amount between +0.1 mm and −0.1 mm was added to each depth. This measure of locomotion signaling was also used to plot the relationship between locomotion signaling and reward responses in Extended Data Fig. 1i (for reward response calculation, see ‘Analysis of responses to rewards and air puffs’ subsection below) and to sort the Δ*F*/*F*-acceleration correlation plots in Extended Data Fig. 1f.

For analysis of timing differences between *Calb1*^+^ and *Vglut2*^+^ deceleration signaling shown in Fig. 2i and Extended Data Fig. 6f,g, the lag between Δ*F*/*F* transient peaks and deceleration peaks was quantified by locating in time the minimum cross-correlated value between 0 s and 1 s for the Δ*F*/*F*-acceleration cross-correlations for each recording (Fig. 2i), the maximum Δ*F*/*F* value between 0 s and 1 s for the triggered average on deceleration (Extended Data Fig. 6f) or the minimum acceleration value between −1 s and 0 s for the triggered average on transient peaks (Extended Data Fig. 6g).

For calculating the relationship between velocity and Δ*F*/*F* as shown in Extended Data Fig. 7c, we divided the velocity and Δ*F*/*F* traces based on the velocity at each timepoint into bins of 0.1 m s^−1^ ((−0.05:0.1:0.75 inf)) and averaged the Δ*F*/*F* for each subtype and bin.

For checking whether the locomotion signaling observed in DAT mice across depths could be explained by mixtures of the *Anxa1*^+^ and *Calb1*^*+*^ subtypes (Extended Data Fig. 6h,h′), we first divided DAT recordings made in the anterior striatum (anterior to bregma) based on the depth from the brain surface at which they were made, from 1.5 mm to 4 mm in 0.5-mm bins, and obtained the average cross-correlation between Δ*F*/*F* and acceleration for each subset (H), as explained above. We then calculated weighted averages between the average cross-correlations for the *Calb1+* and *Anxa1*^+^ subtypes in different ratios to match the approximate relative abundance of each subtype’s axons across depths: from 100% *Anxa1*^+^, 0% *Calb1*^+^ for dorsal striatum to 0% *Anxa1*^+^, 100% *Calb1*^+^ for ventral striatum (H′).

For determining whether the size of the Δ*F*/*F* response scaled with the size of the acceleration/deceleration (Fig. 5a,b), for each recording we divided all the accelerations/decelerations that fulfilled the requirements described above (for triggered averages on accelerations and decelerations) into five quartiles per recording based on the peak acceleration/deceleration and calculated the average acceleration (Fig. 5a, left) and Δ*F*/*F* (Fig. 5a, right) triggered on accelerations/decelerations within each of these five quartiles. For plotting the transient amplitude for each of these acceleration/deceleration quantiles (Fig. 5b), we calculated the difference between the Δ*F*/*F* value at *t* = 0 (trigger point, start of the acceleration/deceleration) and the maximum Δ*F*/*F* value within the 1-s window following it. The fold increase as reported in the legend was calculated by dividing the transient amplitude for the largest deceleration/acceleration quintile by the transient amplitude for the smallest deceleration/acceleration quintile. Statistical significance was calculated using a paired Wilcoxon signed-rank test with Bonferroni correction (*P* values multiplied by 3) comparing the transient amplitude for the smallest versus largest acceleration/deceleration quintiles for each recording.

PCA was applied to the matrix of all cross-correlation traces from striatal recordings (shown in Fig. 2f), from all functionally homogeneous subtypes (*Vglut2*^+^, *Calb1*^+^ and *Anxa1*^+^), using MATLAB’s pca function without centering: ‘centered’, ‘off’. Centering was not used so as to maintain the cross-correlation values’ relationship to 0 and to avoid biasing the results based on the relative number of recordings from different subtypes; however, equivalent results were obtained when we repeated the PCA analysis with centering (Extended Data Fig. 7a). This pca function outputs the PCs (loadings and eigenvectors), the scores for each recording’s cross-correlation along each PC (matrix of all SNc cross-correlation traces multiplied by the loadings matrix) and the variance explained by each PC across all recordings. For the representation of combinations of the first two PCs (PC1 and PC2) shown in Fig. 2j, PC1 and PC2 were weighted by the s.d. of their scores across recordings (~1 for PC1 and ~0.7 for PC2), to accurately represent each quadrant in Fig. 2k,l and Extended Data Figs. 6e and 9k. Figure 2k and Extended Data Fig. 6e show the PC1 and PC2 scores for each recording of each subtype. In Extended Data Fig. 6e, recordings were color-coded based on the depth from brain surface at which they were recorded, as measured by the micromanipulator used to move the fiber during photometry. For Extended Data Fig. 7b, the cross-correlation traces were first normalized by dividing the trace by its absolute maximum value with its sign, so that its lowest value was –1 or its maximum value was +1 while maintaining 0, before PCA analysis. Because PC1 and PC2 explain most of the variance, this results in data points being pushed to a ring around the origin.

For SNc recordings, the cross-correlations between Δ*F*/*F* and acceleration for all recordings of all subtypes, as shown in Extended Data Fig. 9k, were decomposed using the same PCs calculated above from the striatal cross-correlations. Scores for SNc cross-correlations (Extended Data Fig. 9h) were calculated by multiplying the matrix of all SNc cross-correlation traces by the striatal loadings matrix (PCs). The percent of SNc variance explained by each PC (PC1 = 53.2% of variance, PC2 = 24.3%) was calculated as the variance without the mean subtracted (not centered).

In the PC1/PC2 space shown in Fig. 2k, the angle of each point from the origin represents the shape of the cross-correlation between acceleration and Δ*F*/*F* and, thus, the different relationships between subtypes’ signaling and acceleration, whereas the distance from the origin represents the amplitude of the cross-correlation. To quantify the shape of the cross-correlation across subtypes, we calculated the angle of each recording in the PC1/PC2 space (with each PC weighted by its s.d.) and plotted it in a radial histogram (Fig. 2l). This angle was also used for plotting of subtypes in Fig. 6a,b and Extended Data Fig. 9f,g. All angles in this paper are reported as standard with 0° set between quadrants I and IV and angles increasing in the counterclockwise direction (that is, up is 90°). *P* values for reporting statistical significance for the difference between subtypes across this angle PC1/PC2 space were calculating by opening the angular space at 45° (the region where the least recordings from *Calb1*^+^/*Vglut2*^+^/*Anxa1*^+^ fall) and using a Wilcoxon rank-sum test with Bonferroni correction (multiply *P* values by 3) to compare subtypes. This angle was also used to sort cross-correlation and triggered average heat maps in Fig. 2f–h and Extended Data Figs. 6b–d and 9h–j, starting by the middle of the quadrant opposite to the center of mass for each subtype (315° for *Vglut2*^+^, 45° for *Calb1*^+^ and *DAT*^+^ and 135° for *Anxa1*^*+*^ and *Aldh1a1*^+^) and going counterclockwise. Figure 3b and Extended Data Fig. 6a show the anatomical location of each recording color-coded based on the PC1/PC2 angle and distance from the origin for that recording. The colormap was defined by assigning a different color to the middle of each quadrant (45°, 135°, 225° and 315°), where the center of mass of each subtype approximately falls at, and then fading that color to white as the values of PC1 and PC2 decrease to 0. In Fig. 3b (but not in Extended Data Fig. 6a), recording locations were collapsed into a single brain slice for anterior striatum and another for posterior striatum, and locations were shifted a random amount between ±0.4 mm mediolaterally for visibility. For details on how the *x*–*y*–*z* coordinates for each recording were calculated, see the ‘Fiber placement localization’ subsection.

To calculate the difference in locomotion signaling between pairs of recordings based on the distance between them, as shown in Extended Data Fig. 6j, we used the difference between the PC1/PC2 angles of each pair of recordings calculated as above (maximum angle difference is 180°). For the distance between each pair’s recording locations, we used the Euclidian distance between the *x*–*y*–*z* coordinates of the recordings, obtained as described in the ‘Fiber placement localization’ subsection of these methods below. For within-subtype comparisons (*Calb1*^+^, *Anxa1*^+^ and *Vglut2*^+^) and for *DAT*, all recordings for that subtype/*DAT* were compared with all other recordings from that same subtype/DAT. For the mismatch–subtype comparisons, each recording from *Calb1*^+^, *Anxa1*^+^ and *Vglut2*^*+*^ was compared to all recordings from the other two subtypes (for example, each *Calb1*^+^ recording was compared to each *Anxa1*^+^ and *Vglut2*^*+*^ recording). Statistical significance was calculated using a Mann–Whitney *U*-test with Bonferroni correction (*P* values multiplied by 21, the total number of comparisons performed) comparing all the mismatch–subtype pairs with all the *Vglut2*^+^, *Calb1*^+^ or *Anxa1*^*+*^ pairs.

### Analysis of responses to rewards and air puffs

Recordings included for reward and air puff analysis come from 12 *Vglut2*^*+*^ mice (five males, six females, one unrecorded), eight *Calb1*^*+*^ mice (five males, three females), eight *Anxa1*^*+*^ mice (three males, five females), eight *Aldh1a1*^*+*^ mice (four males, three females, one unrecorded) and 12 *DAT+* mice (four males, six females, two unrecorded).

Reward delivery times were only included when the mice consumed the reward (detected by the lick sensor) within a 1-s window from delivery. For analysis of rewards delivered at rest, rewards were excluded if there were any accelerations greater than 2.5 m s^−^^2^ (or decelerations greater than −2.5 m s^−^^2^) in a window of 0.75 s before or after the reward delivery or any accelerations greater than 1.5 m s^−^^2^ (or decelerations greater than −1.5 m s^−^^2^) within a 0.4-s window after the reward (where responses to rewards are detected). Triggered averages on rewards (Figs. 4d,e,l, left, and 5c and Extended Data Figs. 1g, 8a,b,h and 9a), air puffs (Figs. 4f,l, right, and 5e and Extended Data Figs. 8f,i and 9b) and rewards at rest (Extended Data Fig. 8e) were calculated by averaging normalized Δ*F*/*F* traces (or licking traces for Fig. 4e and Extended Data Fig. 8b) in a 1-s window before and after included reward or air puff delivery times. Averages per mouse (Extended Data Fig. 8h,i) were obtained by averaging together the triggered averages for all recordings made from the same mouse.

For plotting of triggered averages above, traces were smoothed over five time-lag bins (0.05 s). Shaded areas represent the mean ± s.e.m. across recordings, and accompanying heat maps show triggered averages for all individual recordings. Heat maps in Fig. 4d–f and Extended Data Figs. 1g, 8a–c and 9a,b were sorted by reward response size (see below). Triggered averages were the result of averaging 20 ± 9 rewards and 12 ± 4 air puffs (mean ± s.d.) per recording.

To calculate the size of the response to each stimulus (change in fluorescence) shown in Figs. 4g–j, 5d,f and 6a,b and Extended Data Figs. 1i, 8d,f,j,k and 9c–g, we calculated the difference between the cumulative fluorescence in a 0.5-s window after each reward or air puff delivery time (+0.05 s to +0.55 s) and the cumulative fluorescence in a 0.5-s window before each reward or air puff delivery time (−0.5 s to 0 s). The response to reward or air puff is defined as the average of this value for all reward or air puff delivery times in a recording. The response to rewards calculated in this manner was used to sort all reward and air puff triggered average heat maps in Fig. 4d–f and Extended Data Figs. 1g, 8a–c,e and 9a,b. Heat maps for air puff responses (Fig. 4f and Extended Data Figs. 8c and 9b) were sorted by the corresponding reward responses for each recording, with recordings with no rewards being shown at the top (mice not licking for certain recordings result in a higher number of recordings included for air puff than reward analysis). Figure 4j,k and Extended Data Fig. 8j,k show the location of each recording color-coded based on the reward or air puff response for that recording, calculated in this manner. In Fig. 4j,k (but not in Extended Data Fig. 8j,k), recording locations were collapsed into a single brain slice for anterior striatum and another for posterior striatum, and locations were shifted a random amount between ±0.4 mm mediolaterally for visibility. For details on how the *x*–*y*–*z* coordinates for each recording were calculated, see the ‘Fiber placement localization’ subsection. For Extended Data Fig. 8g, we mininum–maximum scaled the reward and air puff triggered Δ*F*/*F* traces (subtract the trace minimum value and then divide by the trace maximum, so that the new trace goes from 0 to 1) before calculating the integral as above. Although we expected that this would abolish the differences between *Vglut2*^*+*^ and *Calb1*^+^, it did not, due to GCaMP’s decay not scaling linearly with amplitude.

*P* values for reporting statistical significance for each subtype’s responses to rewards and air puffs (Fig. 4g and Extended Data Fig. 9c) used a non-parametric statistical test (Wilcoxon signed-rank test) with Bonferroni correction (*P* values multiplied by 4). *P* values for reporting sensitivity to reward size (Fig. 2i and Extended Data Fig. 9e) were calculated using a non-parametric paired statistical test (Wilcoxon signed-rank test), with Bonferroni correction (*P* values multiplied by 4), between the responses to small and large rewards in the same recording.

For calculating whether the size of the deceleration accompanying reward and air puff delivery affects the size of each subtype’s response, we split the rewards or air puff delivery events in each recording in two halves based on the size of the deceleration that followed the stimulus, calculated as the integral of the acceleration trace within a 0.75-s window after the stimulus delivery. This strategy was used instead of setting a deceleration threshold for the split so that the same number of events was used within each recording for the large versus small averages. Because of this, large versus small decelerations do not look the same across subtypes (some subtypes had greater decelerations on average). A paired Wilcoxon signed-rank test was used to compare the amplitude of the reward or air puff response with smaller versus larger decelerations, with Bonferroni correction.

SNc recordings (Extended Data Fig. 9a–g) were analyzed in the same manner as striatal recordings. Recordings included for reward and air puff analysis in SNc come from nine *Vglut2*^*+*^ mice (five males, three females, one unrecorded), five *Calb1*^*+*^ mice (three males, two females), six *Anxa1*^*+*^ mice (two males, four females), 11 *Aldh1a1*^*+*^ mice (four males, six females, one unrecorded) and eight *DAT*^*+*^ mice (two males, three females, three unrecorded).

### *k*-means clustering

*k*-means clustering was run using the MATLAB kmeans function for three clusters on the values of reward and air puff responses (see previous section for calculation) and the scores along the first two PCs (PC1 and PC2) from the PCA analysis on cross-correlations between Δ*F*/*F* and acceleration traces (as in Fig. 2k), for all axonal recordings from *Calb1*^+^, *Vglut2*^*+*^ and *Anxa1*^+^ subtypes where all measures were obtained (mice were running above threshold and received rewards and aversive stimuli, following the same inclusion criteria described above in each corresponding section). Data were normalized before *k*-means analysis using the MATLAB ‘normalize’ function, which returns the *z*-score of each variable across recordings with center 0 and s.d. 1. From the three resulting clusters, each subtype was matched to the cluster with the greatest overlap (each cluster was matched to a different subtype), and accuracy was calculated as the percentage of recordings classified within that cluster (Fig. 6c). Because this *k*-means clustering was run on a four-dimensional dataset (reward, air puff, locomotion PC1 score and locomotion PC2 score), Fig. 6a,b instead shows the combination of PC1 and PC2 scores as an angle, as calculated above.

### Cross-correlation between SNc and striatum Δ*F*/*F* traces

Recordings included for comparison between SNc and striatal signaling come from four *Vglut2*^*+*^ mice (three males, one female), two *Calb1*^*+*^ mice (two males), eight *Anxa1*^*+*^ mice (four males, four females), nine *Aldh1a1*^*+*^ mice (four males, four femles, one unrecorded) and five *DAT*^*+*^ mice (two males, three females).

All simultaneously recorded pairs of SNc/striatum recordings where both traces had a signal-to-noise ratio above 10 were included, regardless of behavior. Cross-correlations between SNc and striatum Δ*F*/*F* traces were calculated using MATLAB’s crosscorr function over a 1-s time-lag window (100 bins). For the isosbestic control cross-correlation shown in Fig. 7d,e and Extended Data Fig. 10b,c, we calculated the cross-correlations between SNc-470 and striatum-405 Δ*F*/*F* traces and also between SNc-405 and striatum-470 Δ*F*/*F* traces and averaged the resulting cross-correlation traces together. Any pairs of recordings with a peak 405/470 average cross-correlation above 0.12 were excluded.

For plotting in Fig. 7d,e and Extended Data Fig. 10b,c, 405-nm and 470-nm cross-correlations were smoothed over five bins (0.05 s). Shaded areas in represent the mean ± s.e.m., and accompanying heat maps show cross-correlations for all recordings. For comparison of peak cross-correlations between each subtype and DAT (Fig. 7f and Extended Data Fig. 10d), we used a non-parametric statistical test for two independent populations (Mann–Whitney *U*-test, also called the Wilcoxon rank-sum test), with Bonferroni correction (*P* values were multiplied by the number of comparisons performed).

### Fiber placement localization

For the representation of recording locations in striatum shown in Figs. 2e, 3a,b and 4j–l and Extended Data Figs. 6a and 8j,k, ×20 magnification images of striatum were acquired on a Keyence slide scanner (BZ-X810) (see ‘Methods details, Histology’). For the slice in each brain with the clearest fiber track, fiber tracks were marked onto the images. We then identified the closest reference slice for each imaged brain slice (reference slices from the Paxinos Mouse Brain Atlas), spaced 0.36 mm (bregma +0.86, +0.50, +0.14, −0.22, −0.58, −0.94 and −1.34 mm, as shown in schematics in Extended Data Fig. 6a) and uniformly scaled this reference to approximately match the imaged slice. Recording locations for recordings included in each figure for each mouse were then marked on each slice, measuring depth from brain surface along the fiber track. Circles represent approximate light collection recording area for all recordings given our 200-µm fibers (~300 µm in diameter, estimated based on ref. ^72^). For compact representation in Figs. 3a,b and 4j,k, all slices from anterior striatum (bregma +0.85 to +0.14) or posterior striatum (bregma −0.58 to −1.34) were approximately aligned and combined into a single slice, and recording locations were randomly shifted ±0.4 mm mediolaterally to reduce overlap and improve visibility. The same data but un-collapsed and un-shifted are shown in Extended Data Figs. 6a and 8j. *x*–*y*–*z* coordinates were obtained for each recording using bregma as a reference point (0,0,0), as shown in the Paxinos Mouse Brain Atlas and used for calculating distances between pairs of recordings in Extended Data Fig. 6j. Images of brain slices used for fiber placement localization have been deposited in Zenodo^73^ (10.5281/zenodo.7908382).

### Reporting summary

Further information on research design is available in the Nature Portfolio Reporting Summary linked to this article.

## Online content

Any methods, additional references, Nature Portfolio reporting summaries, source data, extended data, supplementary information, acknowledgements, peer review information; details of author contributions and competing interests; and statements of data and code availability are available at 10.1038/s41593-023-01401-9.

### Supplementary information

Reporting Summary

Supplementary Table 1: key resources.

### Source data

Source Data Fig. 1 Statistical source data.

Source Data Fig. 2 Statistical source data.

Source Data Fig. 3 Statistical source data.

Source Data Fig. 4 Statistical source data.

Source Data Fig. 5 Statistical source data.

Source Data Fig. 6 Statistical source data.

Source Data Fig. 7 Statistical source data.

Source Data Extended Data Fig. 1 Statistical source data.

Source Data Extended Data Fig. 5 Statistical source data.

Source Data Extended Data Fig. 6 Statistical source data.

Source Data Extended Data Fig. 7 Statistical source data.

Source Data Extended Data Fig. 8 Statistical source data.

Source Data Extended Data Fig. 9 Statistical source data.

Source Data Extended Data Fig. 10 Statistical source data.

## Data Availability

Datasets generated in this study have been deposited online and are publicly available as of the date of publication. Raw fiber photometry data have been deposited in Zenodo (10.5281/zenodo.7871634 and https://zenodo.org/record/7871634) as well as the pre-processed dataset for easier access (10.5281/zenodo.7871982 and https://zenodo.org/record/7871982). Raw snRNA-seq data have been deposited to the Gene Expression Omnibus (GEO) (GSE222558). Mouse lines generated in this study will be shared upon reasonable request and upon completion of a material transfer agreement as per institutional policy and will be deposited to a mouse repository (for example, the Mutant Mouse Resource and Research Center). Other datasets and resources used in this manuscript are the Paxinos Mouse Brain Altas book (see Supplementary Table 1 for a link to GoodleBooks), the Allen Mouse Brain Atlas (https://mouse.brain-map.org/) and existing scRNA-seq datasets from Saunders et al. (http://dropviz.org/), Tiklova et al. (GEO: GSE116138), Kramer et al. (GEO: GSE115070) and La Manno et al. (LaMannoBrainData() Command, https://bioconductor.org/packages/release/data/experiment/html/scRNAseq.html). Source data are provided with this paper.

## Code availability

All original code used for analysis of fiber photometry data and snRNA-seq data is available at GitHub (https://github.com/DombeckLab/Azcorra2023/) and has been deposited in Zenodo (10.5281/zenodo.7900531 and https://zenodo.org/record/7900531) and is publicly available as of the date of publication.

## References

[1] Schultz W (1999). The reward signal of midbrain dopamine neurons. N. Physiol. Sci..

[2] Eshel, N., Tian, J., Bukwich, M. & Uchida, N. Dopamine neurons share common response function for reward prediction error. *Nat. Neurosci.* **19**, 479–486 (2016). 10.1038/nn.4239 PMC4767554 26854803

[3] Dabney, W. et al. A distributional code for value in dopamine-based reinforcement learning. *Nature* **577**, 671–675 (2020). 10.1038/s41586-019-1924-6 PMC7476215 31942076

[4] Coddington LT, Dudman JT (2019). Learning from action: reconsidering movement signaling in midbrain dopamine neuron activity. Neuron.

[5] Berridge KC (2007). The debate over dopamine’s role in reward: the case for incentive salience. Psychopharmacolgy (Berl)..

[6] Engelhard, B. et al. Specialized coding of sensory, motor and cognitive variables in VTA dopamine neurons. *Nature* **570**, 509–513 (2019). 10.1038/s41586-019-1261-9 PMC7147811 31142844

[7] de Jong JW (2019). A neural circuit mechanism for encoding aversive stimuli in the mesolimbic dopamine system. Neuron.

[8] Brischoux, F., Chakraborty, S., Brierley, D. I. & Ungless, M. A. Phasic excitation of dopamine neurons in ventral VTA by noxious stimuli. *Proc. Natl Acad. Sci. USA* **106**, 4894–4899 (2009). 10.1073/pnas.0811507106 PMC2660746 19261850

[9] Menegas, W., Babayan, B. M., Uchida, N. & Watabe-Uchida, M. Opposite initialization to novel cues in dopamine signaling in ventral and posterior striatum. *eLife* **6**, e21886 (2017). 10.7554/eLife.21886 PMC5271609 28054919

[10] Menegas, W., Akiti, K., Amo, R., Uchida, N. & Watabe-Uchida, M. Dopamine neurons projecting to the posterior striatum reinforce avoidance of threatening stimuli. *Nat. Neurosci*. **21**, 1421–1430 (2018). 10.1038/s41593-018-0222-1 PMC6160326 30177795

[11] Matsumoto M, Hikosaka O (2009). Two types of dopamine neuron distinctly convey positive and negative motivational signals. Nature.

[12] Lerner TN (2015). Intact-brain analyses reveal distinct information carried by SNc dopamine subcircuits. Cell.

[13] Howe MW, Dombeck DA (2016). Rapid signalling in distinct dopaminergic axons during locomotion and reward. Nature.

[14] Da Silva JA (2018). Dopamine neuron activity before action initiation gates and invigorates future movements. Nature.

[15] Fan D, Rossi MA, Yin HH (2012). Mechanisms of action selection and timing in substantia nigra neurons. J. Neurosci..

[16] Coddington LT, Dudman JT (2018). The timing of action determines reward prediction signals in identified midbrain dopamine neurons. Nat. Neurosci..

[17] Schultz W, Ruffieux A, Aebischer P (1983). The activity of pars compacta neurons of the monkey substantia nigra in relation to motor activation. Exp. Brain Res..

[18] Dodson PD (2016). Representation of spontaneous movement by dopaminergic neurons is cell-type selective and disrupted in parkinsonism. Proc. Natl Acad. Sci. USA.

[19] Brown HD, Mccutcheon JE, Cone JJ, Ragozzino ME, Roitman MF (2011). Primary food reward and reward-predictive stimuli evoke different patterns of phasic dopamine signaling throughout the striatum. Eur. J. Neurosci..

[20] Tsutsui-Kimura I (2020). Distinct temporal difference error signals in dopamine axons in three regions of the striatum in a decision-making task. eLife.

[21] Hamid AA, Frank MJ, Moore CI (2021). Wave-like dopamine dynamics as a mechanism for spatiotemporal credit assignment. Cell.

[22] Poulin JF, Gaertner Z, Moreno-Ramos OA, Awatramani R (2020). Classification of midbrain dopamine neurons using single-cell gene expression profiling approaches. Trends Neurosci..

[23] Poulin JF (2014). Defining midbrain dopaminergic neuron diversity by single-cell gene expression profiling. Cell Rep..

[24] La Manno G (2016). Molecular diversity of midbrain development in mouse, human, and stem cells. Cell.

[25] Saunders A (2018). Molecular diversity and specializations among the cells of the adult mouse brain. Cell.

[26] Tiklová K (2019). Single-cell RNA sequencing reveals midbrain dopamine neuron diversity emerging during mouse brain development. Nat. Commun..

[27] Kramer DJ, Risso D, Kosillo P, Ngai J, Bateup HS (2018). Combinatorial expression of *Grp* and *Neurod6* defines dopamine neuron populations with distinct projection patterns and disease vulnerability. eNeuro.

[28] Poulin J-FF (2018). Mapping projections of molecularly defined dopamine neuron subtypes using intersectional genetic approaches. Nat. Neurosci..

[29] Barter JW (2015). Beyond reward prediction errors: the role of dopamine in movement kinematics. Front. Integr. Neurosci..

[30] Tian L (2009). Imaging neural activity in worms, flies and mice with improved GCaMP calcium indicators. Nat. Methods.

[31] Lee SJ (2021). Cell-type-specific asynchronous modulation of PKA by dopamine in learning. Nature.

[32] Patriarchi, T. et al. An expanded palette of dopamine sensors for multiplex imaging in vivo. *Nat. Methods* **17**, 1147–1155 (2020). 10.1038/s41592-020-0936-3 PMC8169200 32895537

[33] Cachope R (2012). Selective activation of cholinergic interneurons enhances accumbal phasic dopamine release: setting the tone for reward processing. Cell Rep..

[34] Liu C (2022). An action potential initiation mechanism in distal axons for the control of dopamine release. Science.

[35] Liu G (2014). Aldehyde dehydrogenase 1 defines and protects a nigrostriatal dopaminergic neuron subpopulation. J. Clin. Invest..

[36] Pereira Luppi M (2021). *Sox6* expression distinguishes dorsally and ventrally biased dopamine neurons in the substantia nigra with distinctive properties and embryonic origins. Cell Rep..

[37] Hobson BD (2022). Subcellular and regional localization of mRNA translation in midbrain dopamine neurons. Cell Rep..

[38] Grindberg RV (2013). RNA-sequencing from single nuclei. Proc. Natl Acad. Sci. USA.

[39] Kamath, T. et al. A molecular census of midbrain dopaminergic neurons in Parkinson’s disease. Preprint at *bioRxiv*10.1101/2021.06.16.448661 (2021).

[40] Tobler PN, Fiorillo CD, Schultz W (2005). Adaptive coding of reward value by dopamine neurons. Science.

[41] Ding JB, Guzman JN, Peterson JD, Goldberg JA, Surmeier DJ (2010). Thalamic gating of corticostriatal signaling by cholinergic interneurons. Neuron.

[42] Threlfell S (2012). Striatal dopamine release is triggered by synchronized activity in cholinergic interneurons. Neuron.

[43] Mohebi A (2019). Dissociable dopamine dynamics for learning and motivation. Nature.

[44] Kim HGR (2020). A unified framework for dopamine signals across timescales. Cell.

[45] Schultz W (1998). Predictive reward signal of dopamine neurons. J. Neurophysiol..

[46] Lahiri AAK, Bevan MD (2020). Dopaminergic transmission rapidly and persistently enhances excitability of D1 receptor-expressing striatal projection neurons. Neuron.

[47] Wu J (2019). Distinct connectivity and functionality of aldehyde dehydrogenase 1a1-positive nigrostriatal dopaminergic neurons in motor learning. Cell Rep..

[48] Saunders BT, Richard JM, Margolis EB, Janak PH (2018). Dopamine neurons create Pavlovian conditioned stimuli with circuit-defined motivational properties. Nat. Neurosci..

[49] Markowitz JE (2023). Spontaneous behaviour is structured by reinforcement without explicit reward. Nature.

[50] Patriarchi, T. et al. Ultrafast neuronal imaging of dopamine dynamics with designed genetically encoded sensors. *Science* **360**, eaat4422 (2018). 10.1126/science.aat4422 PMC6287765 29853555

[51] Zell V (2020). VTA glutamate neuron activity drives positive reinforcement absent dopamine co-release. Neuron.

[52] Melani R, Tritsch NX (2022). Inhibitory co-transmission from midbrain dopamine neurons relies on presynaptic GABA uptake. Cell Rep..

[53] Kim, J. I. et al. Aldehyde dehydrogenase 1a1 mediates a GABA synthesis pathway in midbrain dopaminergic neurons. *Science* **350**, 102–106 (2015). 10.1126/science.aac4690 PMC4725325 26430123

[54] Tritsch NX, Ding JB, Sabatini BL (2012). Dopaminergic neurons inhibit striatal output through non-canonical release of GABA. Nature.

[55] Howe, M. et al. Coordination of rapid cholinergic and dopaminergic signaling in striatum during spontaneous movement. *eLife* **8**, e44903 (2019). 10.7554/eLife.44903 PMC6457892 30920369

[56] Fu Y, Paxinos G, Watson C, Halliday GM (2016). The substantia nigra and ventral tegmental dopaminergic neurons from development to degeneration. J. Chem. Neuroanat..

[57] Kish SJ, Shannak K, Hornykiewicz O (1988). Uneven pattern of dopamine loss in the striatum of patients with idiopathic Parkinson’s disease. N. Engl. J. Med..

[58] Sgobio C (2017). Aldehyde dehydrogenase 1-positive nigrostriatal dopaminergic fibers exhibit distinct projection pattern and dopamine release dynamics at mouse dorsal striatum. Sci. Rep..

[59] Steinkellner T (2022). Dopamine neurons exhibit emergent glutamatergic identity in Parkinson’s disease. Brain.

[60] Gaertner Z, Azcorra M, Dombeck DA, Awatramani R (2022). Molecular heterogeneity in the substantia nigra: a roadmap for understanding PD motor pathophysiology. Neurobiol. Dis..

[61] Gaertner, Z. Anxa1-iCre and Aldh1a1-iCre raw image source data for Azcorra, Gaertner et al. 10.5281/zenodo.7909331 (2023).

[62] Azcorra, M. Midbrain viral injections for striatal fiber photometry in mice. 10.17504/protocols.io.5qpvor8zxv4o/v1 (2023).

[63] Azcorra, M. Acute striatal or midbrain fiber photometry in head-fixed mice. 10.17504/protocols.io.4r3l27yj4g1y/v1 (2023).

[64] Azcorra, M. & Gaertner, Z. DombeckLab/Azcorra2023: Azcorra et al. Nature Neuro 2023 code. 10.5281/zenodo.7900531 (2023).

[65] Hafemeister C, Satija R (2019). Normalization and variance stabilization of single-cell RNA-seq data using regularized negative binomial regression. Genome Biol..

[66] Welch JD (2019). Single-cell multi-omic integration compares and contrasts features of brain cell identity. Cell.

[67] Choudhary, S. & Satija, R. Comparison and evaluation of statistical error models for scRNA-seq. *Genome Biol*. **23**, 27 (2022). 10.1186/s13059-021-02584-9 PMC8764781 35042561

[68] Linderman GC (2022). Zero-preserving imputation of single-cell RNA-seq data. Nat. Commun..

[69] Lein ES (2006). Genome-wide atlas of gene expression in the adult mouse brain. Nature.

[70] Azcorra, M. (2023). Azcorra2023—raw fiber photometry recordings. 10.5281/zenodo.7871634 (2023).

[71] Azcorra, M. Azcorra2023—fiber photometry recordings (pre-processed to get DF/F). 10.5281/zenodo.7871982 (2023).

[72] Pisanello M (2019). The three-dimensional signal collection field for fiber photometry in brain tissue. Front. Neurosci..

[73] Azcorra, M. Azcorra2023—histological images for fiber placement localization. 10.5281/zenodo.7908382 (2023).

