# Supplementary material for: Unique functional responses differentially map onto genetic subtypes of dopamine neurons
Source: Nat Neurosci. 2023 Aug 3;26(10):1762–74. doi: 10.1038/s41593-023-01401-9 (PMC10545540; doi:10.1038/s41593-023-01401-9)
Supplement: Supplementary file 1 — Reporting Summary [file 41593_2023_1401_MOESM1_ESM.pdf]

nature portfolio

Corresponding author(s): Daniel A Dombeck, Rajeshwar Awatramani

Last updated by author(s): May 8, 2023

# Reporting Summary

Nature Portfolio wishes to improve the reproducibility of the work that we publish. This form provides structure for consistency and transparency in reporting. For further information on Nature Portfolio policies, see our Editorial Policies and the Editorial Policy Checklist.

## Statistics

For all statistical analyses, confirm that the following items are present in the figure legend, table legend, main text, or Methods section.

| n/a | Confirmed | |
|---|---|---|
| ☐ | ☒ | The exact sample size ($n$) for each experimental group/condition, given as a discrete number and unit of measurement |
| ☐ | ☒ | A statement on whether measurements were taken from distinct samples or whether the same sample was measured repeatedly |
| ☐ | ☒ | The statistical test(s) used AND whether they are one- or two-sided<br>*Only common tests should be described solely by name; describe more complex techniques in the Methods section.* |
| ☒ | ☐ | A description of all covariates tested |
| ☐ | ☒ | A description of any assumptions or corrections, such as tests of normality and adjustment for multiple comparisons |
| ☐ | ☒ | A full description of the statistical parameters including central tendency (e.g. means) or other basic estimates (e.g. regression coefficient) AND variation (e.g. standard deviation) or associated estimates of uncertainty (e.g. confidence intervals) |
| ☐ | ☒ | For null hypothesis testing, the test statistic (e.g. $F$, $t$, $r$) with confidence intervals, effect sizes, degrees of freedom and $P$ value noted<br>*Give P values as exact values whenever suitable.* |
| ☒ | ☐ | For Bayesian analysis, information on the choice of priors and Markov chain Monte Carlo settings |
| ☒ | ☐ | For hierarchical and complex designs, identification of the appropriate level for tests and full reporting of outcomes |
| ☐ | ☒ | Estimates of effect sizes (e.g. Cohen's $d$, Pearson's $r$), indicating how they were calculated |

*Our web collection on statistics for biologists contains articles on many of the points above.*

## Software and code

Policy information about availability of computer code

| | |
|---|---|
| Data collection | Picoscope6, Labview 2011, and Olympus VS-ASW-S6 and cellSens |
| Data analysis | MATLAB R2021a, ImageJ 1.48v, R version 4.0.2, RStudio Version 1.2.5042, Seurat R Package Versions 3.2.0 and 4.0.2, LIGER R Package 1.0.0, scRNAseq R Package 2.2.0, SeuratWrappers 0.2.0 R Package, BD FACSDiva 8.0.3. Custom code at GitHub: https://github.com/DombeckLab/Azcorra2023/ (DOI: 10.5281/zenodo.7900531) |

For manuscripts utilizing custom algorithms or software that are central to the research but not yet described in published literature, software must be made available to editors and reviewers. We strongly encourage code deposition in a community repository (e.g. GitHub). See the Nature Portfolio guidelines for submitting code & software for further information.

## Data

Policy information about availability of data

All manuscripts must include a data availability statement. This statement should provide the following information, where applicable:

- Accession codes, unique identifiers, or web links for publicly available datasets
- A description of any restrictions on data availability
- For clinical datasets or third party data, please ensure that the statement adheres to our policy

NEW DATASETS. Fiber photometry raw data: https://zenodo.org/record/7871634, DOI 10.5281/zenodo.7871634. Pre-processed data (DF/F) and metadata: https://zenodo.org/record/7871982, DOI 10.5281/zenodo.7871982. Single Nucleus RNASeq Raw Data: GEO GSE222558.
EXISTING DATASETS. Paxinos Mouse Brain atlas book. Allen Mouse Brain Atlas: https://mouse.brain-map.org/. Saunders et al. RNAseq data: http://dropviz.org/. Tiklova et al. RNAseq data: GEO: GSE116138. Kramer et al. RNAseq data: GEO: GSE115070. La Manno et al. RNAseq data: https://bioconductor.org/packages/release/data/experiment/html/scRNAseq.html.

## Human research participants

Policy information about studies involving human research participants and Sex and Gender in Research.

| | |
|---|---|
| Reporting on sex and gender | N/A |
| Population characteristics | N/A |
| Recruitment | N/A |
| Ethics oversight | N/A |

Note that full information on the approval of the study protocol must also be provided in the manuscript.

# Field-specific reporting

Please select the one below that is the best fit for your research. If you are not sure, read the appropriate sections before making your selection.

☒ Life sciences ☐ Behavioural & social sciences ☐ Ecological, evolutionary & environmental sciences

For a reference copy of the document with all sections, see nature.com/documents/nr-reporting-summary-flat.pdf

# Life sciences study design

All studies must disclose on these points even when the disclosure is negative.

| | |
|---|---|
| Sample size | No statistical methods were used to pre-determine sample sizes, but our sample sizes are similar to those reported in previous publications (Howe and Dombeck, Nature 2016; Da Silva et al., Nature 2018; Coddington & Dudman, Nat. Neurosci. 2018). |
| Data exclusions | A whole section of the methods is dedicated to explaining excluding criteria in detail. In brief, fiber photometry recordings were excluded if transient signal-to-noise ratios were below a threshold, which occurred when recordings were made in regions without sufficient axons or somas of the subtype of interest. This threshold was determined independently of the subsequent analysis steps. Recordings were also excluded if movement artifacts were detected in the 405 nm isosbestic control. For behavioral analysis, recordings where mice were not running for at least 100s were excluded from locomotion analysis, and rewards delivery times were the mice did not lick immediately after the reward was delivered were also excluded from analysis. |
| Replication | The new *Anxa1+* subtype was identified from analysis of both a meta-dataset of existing single-cell RNAseq and a new dataset of single-nucleus RNAseq. Subtype marker expression was corroborated using the Allen Brain Atlas in situ hybridization dataset. Locomotion signaling was corroborated using several complimentary analysis (cross-correlation with acceleration and different triggered averages. PCA analysis and reward/air puff signaling were corroborated using different normalization settings. Functional analysis of different subtypes was corroborated in recordings from striatum and SNc. |
| Randomization | The same experiments and measures were made from different dopaminergic subtypes such that other subtypes served as controls to each other. We also repeated the experiments in DAT-Cre mice where all subtypes were simultaneously recorded from, as an additional control. Thus, group randomization was not necessary. |
| Blinding | Data collection and analysis were not performed blind to the conditions of the experiments, as different subtypes require recording from different (though partially overlapping) regions of striatum, due to their different projection regions. Exclusion criteria were selected blind subtype identity and subsequent analysis. |

# Reporting for specific materials, systems and methods

We require information from authors about some types of materials, experimental systems and methods used in many studies. Here, indicate whether each material, system or method listed is relevant to your study. If you are not sure if a list item applies to your research, read the appropriate section before selecting a response.

### Materials & experimental systems

| n/a | Involved in the study | |
|---|---|---|
| ☐ | ☒ | Antibodies |
| ☒ | ☐ | Eukaryotic cell lines |
| ☒ | ☐ | Palaeontology and archaeology |
| ☐ | ☒ | Animals and other organisms |
| ☒ | ☐ | Clinical data |
| ☒ | ☐ | Dual use research of concern |

### Methods

| n/a | Involved in the study | |
|---|---|---|
| ☒ | ☐ | ChIP-seq |
| ☐ | ☒ | Flow cytometry |
| ☒ | ☐ | MRI-based neuroimaging |

## Antibodies

| | |
|---|---|
| Antibodies used | Primaries (dilution 1:1000 except when specified):<br>1. Sheep anti-Tyrosine Hydroxylase (TH) Pel-FreezCat# P60101-0, RRID:AB_461070;<br>2. Rabbit anti-GFP (for GCaMP6f) Thermo Fisher Scientific (Invitrogen) Cat# A-11122, RRID:AB_221569<br>3. Rabbit anti-Anxa1 antibody Thermo Fisher Scientific Cat# 71-3400, RRID:AB_2533983 (1:500)<br>4. Goat anti-Aldh1a1 R&D Systems Cat# AF5869, RRID:AB_2044597 (1:500)<br>5. Mouse anti-Tyrosine Hydroxylase (TH) Sigma-Aldrich Cat# T2928, RRID:AB_477569<br>6. Rabbit anti-Tyrosine Hydroxylase (TH) Pel-Freez Cat# P40101-0, RRID:AB_461064<br>7. Rat anti-mCherry Thermo Fisher Scientific (Invitrogen) Cat# M11217, RRID:AB_2536611 (1:2000)<br>Secondaries (dilution 1:250):<br>8. Donkey anti-Rabbit Alexa Fluor 488 Jackson ImmunoResearch Labs Cat# 711-545-152, RRID:AB_2313584<br>9. Donkey anti-goat Alexa Fluor 488Molecular Probes Cat# A-11055, RRID:AB_2534102<br>10. Donkey anti-mouse Alexa Fluor 647 Thermo Fisher Scientific (Invitrogen) Cat# A-31571, RRID:AB_162542<br>11. Donkey anti-rabbit Alexa Fluor 647Thermo Fisher Scientific (Invitrogen) Cat# A-31573, RRID:AB_2536183<br>12. Donkey anti-rat Cy3Jackson ImmunoResearch Labs Cat# 712-165-153, RRID:AB_2340667<br>13. Donkey anti-Sheep Alexa Fluor 555 Thermo Fisher Scientific (Invitrogen) Cat# A-21436, RRID:AB_2535857 |
| Validation | 1. Sheep anti-Th: Manufacturer's website: "10 ug of rat caudate lysate showing specific immunolabeling of the ~60k TH protein in Western Blot."<br>2. Rabbit anti-GFP: Manufacturer's website "This antibody was verified by Relative expression to ensure that the antibody binds to the antigen stated"<br>3. Rabbit anti-Anxa1: Manufacturer's website "This Antibody was verified by Knockout to ensure that the antibody binds to the antigen stated."<br>4. Goat anti-Aldh1a1: Manufacturer's website "Detects human Aldehyde Dehydrogenase 1-A1/ALDH1A1 in direct ELISAs and detects human and mouse Aldehyde Dehydrogenase 1-A1/ALDH1A1 in Western blots"<br>5. Mouse anti-Th: Manufacturer's website "The antibody is reactive in immunohistology, immunoblotting, and immuno-precipitation protocols and cross-reacts with TH from numerous mammalian species"<br>6. Rabbit anti-Th: Manufacturer's website "Reactivity to all mammalian and at least some non-mammalian forms of the enzyme in Western blots and in IHC/IF"<br>7. Rat anti-mCherry: Manufacturer's website "A ~43 kDa band corresponding to H3-mCherry and 87 kDa band corresponding to p65-RFP were observed in HEK293E transfected lysates on probing with the primary antibody" |

## Animals and other research organisms

Policy information about studies involving animals; ARRIVE guidelines recommended for reporting animal research, and Sex and Gender in Research

| | |
|---|---|
| Laboratory animals | Mouse strains - C57BL6 background, used as adults 2-5 months old:<br>1. Aldh1a1-2A-iCre (new line)<br>2. Anxa1-iCre (new line)<br>3. Calb1-IRES2-Cre, The Jackson Laboratory Strain #:028532,RRID:IMSR_JAX:028532<br>4. VGlut2-IRES-Cre, The Jackson Laboratory Strain #:016963RRID:IMSR_JAX:016963<br>5. DAT-CRE, The Jackson Laboratory Strain #:020080,RRID:IMSR_JAX:020080<br>6. Th-2A-Flpo, from Poulin et al., 2018<br>7. DAT-PF-tTA, The Jackson Laboratory Strain#:027178,RRID:IMSR_JAX:027178<br>8. Ai93D (TITL-GCaMP6f), The Jackson Laboratory Strain #:024107,RRID:IMSR_JAX:024107<br>9. CAG-Sun1/sfGFP, The Jackson Laboratory Strain #:021039,RRID:IMSR_JAX:021039 |
| Wild animals | N/A |
| Reporting on sex | Both males and females were used for all dopaminergic subtypes studied, and no differences between sexes were found. |
| Field-collected samples | N/A |
| Ethics oversight | All animals used in this study were maintained and cared following protocols approved by the Northwestern Animal Care and Use Committee. |

Note that full information on the approval of the study protocol must also be provided in the manuscript.

## Flow Cytometry

### Plots

Confirm that:

☒ The axis labels state the marker and fluorochrome used (e.g. CD4-FITC).

☒ The axis scales are clearly visible. Include numbers along axes only for bottom left plot of group (a 'group' is an analysis of identical markers).

☒ All plots are contour plots with outliers or pseudocolor plots.

☒ A numerical value for number of cells or percentage (with statistics) is provided.

### Methodology

| | |
|---|---|
| Sample preparation | Dat::Cre, Rosa26-LSL-Sun1-sGFP mice were sacrificed and decapitated, after which brains were dissected on ice to extract the ventral midbrain from each animal. Midbrain tissue was dounce homogenized in a nuclear extraction buffer, and washed several times by centrifugation and resuspension in a nuclear resuspension buffer. Each sample was stained with DAPI and thoroughly resuspended for sorting. |
| Instrument | Flow Cytometry Cell Sorting was performed on a BD FACSAria SORP system. |
| Software | BD FACSDiva 8.0.3 |
| Cell population abundance | While not directly quantified, the final sorted fraction appears highly specific for GFP+ nuclei. This is based on two factors: 1) examination of nuclei under a microscope after sorting showed extremely few DAPI+/GFP- nuclei in the sample. Secondly, GFP in this experiment is dependent on DAT expression (i.e. dopaminergic neurons will have GFP+ nuclei), and all final clusters from the downstream RNAseq analyses using the sorted nuclei showed expression of dopamine neuron markers (though notably some clusters had significantly lower levels of these markers). |
| Gating strategy | Gating strategy: First, gates were placed based on FSC-A vs SSC-A in order to roughly separate smaller debris (the vast majority of the input sample) from putative nuclei based on size. This gate was drawn for high sensitivity of nuclei rather than specificity, as later gates would ultimately limit it to GFP+ nuclei. Next, singlet nuclei were selected based on DAPI-W vs DAPI-A, limiting the gates to the main cluster of DAPI+ events while eliminating those with disproportionate DAPI width. Finally, plotting FITC-A vs PerCP-Cy5-5-A showed a very clearly defined population of GFP+ nuclei with drastically more FITC-A than all other events. Final sorting gates were drawn around this distinct population. |

☒ Tick this box to confirm that a figure exemplifying the gating strategy is provided in the Supplementary Information.
